# Cyclophosphamide-Mediated Induction of Myeloid-Derived Suppressor Cells In Vivo: Kinetics of Accumulation, Immune Profile, and Immunomodulation by Oleanane-Type Triterpenoids

**DOI:** 10.3390/ijms27020564

**Published:** 2026-01-06

**Authors:** Mona S. Awad, Aleksandra V. Sen’kova, Andrey V. Markov, Oksana V. Salomatina, Marina A. Zenkova, Oleg V. Markov

**Affiliations:** 1 Institute of Chemical Biology and Fundamental Medicine SB RAS, Academician Lavrentyev Ave. 8, 630090 Novosibirsk, Russia; mona.awad.edu@gmail.com (M.S.A.); senkova_av@1bio.ru (A.V.S.); markov_av@1bio.ru (A.V.M.); marzen@1bio.ru (M.A.Z.); 2 Faculty of Natural Sciences, Novosibirsk State University, Pirogova Str. 2, 630090 Novosibirsk, Russia; 3 N.N. Vorozhtsov Novosibirsk Institute of Organic Chemistry SB RAS, Academician Lavrentyev Ave., 9, 630090 Novosibirsk, Russia; ana@nioch.nsc.ru

**Keywords:** myeloid-derived suppressor cells, MDSCs, immunosuppression, tumor, chemotherapy, CHOP, cyclophosphamide, triterpenoids

## Abstract

Myeloid-derived suppressor cells (MDSCs) are immature myeloid cells that strongly suppress immunity and expand during tumor progression. Various antitumor chemotherapy agents can induce MDSC accumulation, reducing treatment effectiveness. We investigated the impact of the CHOP regimen and its components (cyclophosphamide (CTX), doxorubicin, vincristine, and prednisolone) on the dynamics of MDSC accumulation and the associated changes in immune cell profiles in the peripheral blood and spleen of healthy and lymphosarcoma RLS_40_-bearing CBA mice. CHOP induced significant thymic atrophy and splenomegaly, T-cell depletion, and robust accumulation of MDSCs, primarily polymorphonuclear MDSCs. Kinetic analysis in healthy mice revealed splenic MDSC expansion and T-cell depletion peaked 10-day post-CHOP, driven mainly by CTX; whereas doxorubicin, vincristine, and prednisolone exerted minimal immunological effects. To mitigate CTX-induced MDSCs, glycyrrhizic acid (GLZ), a natural triterpenoid with known immunomodulatory properties, and febroxolone methyl (FM), its novel cyano enone derivative, were administered to CTX-treated mice. GLZ significantly attenuated splenic MDSC accumulation, partially restored T-cell function, and improved immune organ morphology. Conversely, FM exacerbated immunosuppression by expanding MDSCs, enhancing their function by upregulation of *Nos1* and *Ido1* in vivo, and promoting the generation of highly immunosuppressive bone marrow-derived MDSCs in vitro. Thus, our results highlight CTX’s central role in CHOP-induced MDSC expansion. The structure-dependent duality of triterpenoids, countering (GLZ) or promoting (FM) MDSC expansion, offers therapeutic potential for pathologies ranging from chemotherapy-induced side effects to autoimmunity.

## 1. Introduction

Myeloid-derived suppressor cells (MDSCs) are one of the major immunosuppressive factors in tumors and other pathologic conditions associated with chronic inflammation [1,2]. They are a heterogeneous population of immature myeloid cells, closely related to neutrophils and monocytes, that are aberrantly activated by various tumor-derived factors and inflammatory mediators [1,3,4,5,6]. MDSCs are broadly classified into two major subsets: polymorphonuclear CD11b^+^Ly6G^+^Ly6C^low^ PMN-MDSCs, also known as granulocytic MDSCs, and monocytic CD11b^+^Ly6G^−^Ly6C^hi^ M-MDSCs, that employ slightly different mechanisms to suppress T cells [7,8]. In general, MDSCs mediate T-cell inactivation through the following: (1) depletion of essential amino acids with metabolic enzymes arginase-1 (Arg-1), indoleamine 2,3-dioxygenase (IDO), and inducible nitric oxide synthase (iNOS) [2,9,10]; (2) secretion of immunosuppressive interleukin 10 (IL-10) and transforming growth factor beta (TGF-β) [2]; (3) production of reactive oxygen species (ROS) and nitric oxide (NO) [10,11]; (4) programmed death-ligand 1 (PD-L1)-mediated signaling [2]. Furthermore, MDSCs facilitate remodeling of the tumor microenvironment through the release of vascular endothelial growth factor (VEGF) and matrix metalloproteinases [9,12]. Cumulatively, these MDSC-driven factors facilitate tumor evasion from immune surveillance, promoting tumor growth and metastatic dissemination [13,14]. Expansion of MDSCs has been reported in various tumors, including breast [15], prostate [16], lung [17], colorectal [18,19] cancers, melanoma [20,21], hepatocellular carcinoma [22,23], pancreatic ductal adenocarcinoma [24,25,26], glioblastoma [27], and acute myeloid leukemia [28], as well as in corresponding animal tumor models [8].

Polychemotherapy has served as a cornerstone in the treatment of tumors, particularly hematologic malignancies [29]. Among the various polychemotherapeutic regimens used, the CHOP protocol, combining cyclophosphamide (CTX, an alkylating agent), doxorubicin (DOX, a DNA intercalator), vincristine (VCR, an inhibitor of microtubule formation), and prednisolone (PNL, an immunosuppressive corticosteroid), is the most established [30]. First introduced in the early 1980s, the CHOP regimen became the standard first-line treatment for advanced non-Hodgkin lymphoma [31]. It continues to be widely used in clinical practice for the treatment of various other malignancies and has served as the foundation for the development of novel chemotherapeutic protocols [30,32,33].

Substantial experimental evidence demonstrates that chemotherapeutic drugs exert profound and context-dependent effects on MDSCs, making them a double-edged sword in cancer treatment. For instance, gemcitabine was demonstrated to robustly deplete MDSCs in the spleen, blood, and bone marrow in 4T1 mammary carcinoma-bearing mice, enhancing T-cell activity and tumor growth control [34]. Low-dose paclitaxel reduced tumor-associated MDSCs, enhancing anti-tumor immune responses in the melanoma model [35]. 5-Fluorouracil (5-FU) preferentially eliminates tumor-infiltrating MDSCs, augmenting cytotoxic T-cell responses in the EL4 T-cell lymphoma model [36]. Surprisingly, in hepatocellular carcinoma, 5-FU conversely increased MDSC accumulation and reduced immune checkpoint inhibitor efficacy [37]. In the case of CHOP components, cyclophosphamide (CTX) also exerts a biphasic effect on antitumor immunity: when administered at a low dose and specific time points, it can transiently reduce regulatory T-cell (Treg) numbers and suppress their inhibitory capacity [38]. Conversely, moderate to high doses of CTX promoted the expansion and activation of immunosuppressive MDSCs, facilitating tumor evasion from immune surveillance [39]. These findings highlight the critical importance of careful selection of chemotherapeutics, optimization of dosing, and treatment schedules to balance cytotoxic and immunomodulatory outcomes. This rationale motivates further investigation of how the CHOP regimen and its components may modulate MDSC dynamics and function.

Therapeutic strategies targeting MDSCs received growing interest due to their pivotal role in tumor-induced immunosuppression. Among emerging immunomodulators, oleanane-type triterpenoids show significant promise for regulating MDSC function. It was shown that glycyrrhizic acid (GLZ) suppressed pSTAT3 signaling, a pathway critical for the immunosuppressive activity of MDSCs and Tregs, and downregulated cyclooxygenase-2 (COX-2), prostaglandin E2 (PGE2), and Arg1 expression in intratumoral MDSCs, restoring T-cell effector functions [40]. Similarly, synthetic triterpenoid bardoxolone methyl (CDDO-Me) entirely abrogated MDSC-mediated immunosuppression and ROS production, leading to enhanced anti-tumor responses [41]. Previously, it was demonstrated that soloxolone methyl (SM), a cyano enone-bearing GLZ derivative that is structurally related to CDDO-Me, exerts immunomodulatory properties targeting inflammatory myeloid cells [42]. Specifically, SM suppressed inflammatory response by inhibiting ROS and NO production in macrophages, reducing their phagocytic and migration activity via downregulation of NF-κB and Akt signaling [42]. These findings confirm that triterpenoids have great potential to reduce the accumulation and functional activity of MDSCs, thereby potentiating anti-tumor immunity.

In the present study, we investigated the effect of CHOP administration on MDSC expansion in both lymphosarcoma RLS_40_-bearing and healthy mice, evaluated the immunosuppressive properties of its individual components (CTX, DOX, VCR, and PNL), and characterized the dynamics of MDSC accumulation throughout the treatment course. Further, we evaluated the immunomodulatory effects of GLZ and its cyano enone-bearing analog febroxolone methyl (FM), on chemotherapy-induced MDSC expansion with a focus on phenotype, cell numbers, and functional characteristics of MDSCs in vitro and in vivo.

## 2. Results

### 2.1. CHOP Polychemotherapy Induces the Robust Expansion of MDSCs in Blood and Spleen of Mice Irrespective of the Tumor Growth

**Figure 1:**
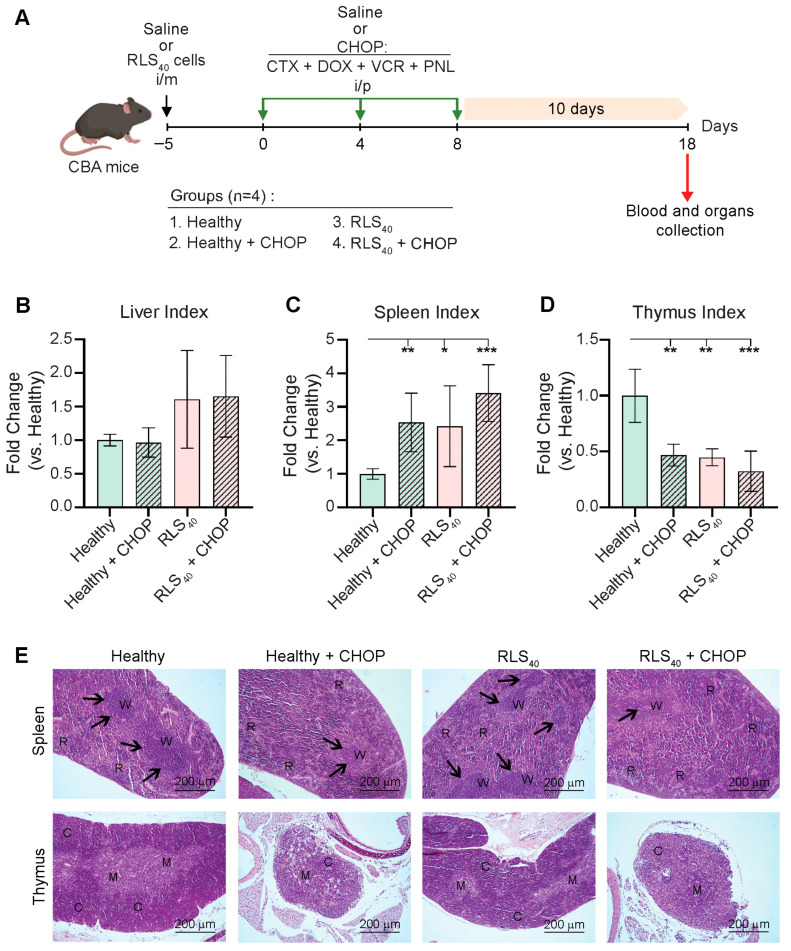
The effects of CHOP polychemotherapy on liver and immune organs in RLS_40_-bearing and healthy CBA mice. (**A**) Experimental setup. Naïve CBA mice were i/m inoculated with RLS_40_ cells or saline to generate healthy or RLS_40_-bearing groups. The mice were then treated i/p with CHOP polychemotherapy according to the experimental schedule. (**B**–**D**) Liver, spleen, and thymus indices represent organ-to-body weight ratios in experimental groups normalized to organ indexes of healthy control. Data from two independent biological experiments (n = 4) are presented as mean ± SD. Statistical analysis was performed by one-way ANOVA with Tukey’s post hoc test. * *p* < 0.05, ** *p* < 0.01, *** *p* < 0.001. (**E**) Representative histological images of spleen and thymus of healthy and tumor-bearing mice after polychemotherapy. Hematoxylin and eosin staining. Original magnification ×100. Black arrows indicate lymphoid follicles in the spleen. R: spleen red pulp, W: spleen white pulp; C: thymus cortex; M: thymus medulla.

Lymphosarcoma RLS_40_-bearing mice received the CHOP regimen starting on day 5 after i/m transplantation of tumor cells. The tumor-bearing mice and healthy animals received three i/p administrations of CHOP at 4-day intervals and were sacrificed on day 23 of tumor development (10 days after the final round of CHOP) (Figure 1A).

As an initial step, we assessed the indices of key organs (liver, spleen, and thymus) in order to evaluate the toxicity and immunomodulatory property of CHOP in both healthy and RLS_40_-bearing mice that either received CHOP or were untreated (Figure 1B–D). The liver indices remained mostly unchanged across the groups, suggesting no significant hepatotoxicity induced by tumor development and CHOP administration (Figure 1B). In contrast, striking alterations were observed in immune organ indices. The spleen indices were significantly altered in both RLS_40_-bearing and CHOP-treated groups compared to healthy controls, indicating pronounced splenic enlargement (Figure 1C). This effect was most prominent in RLS_40_-bearing mice that received CHOP (Figure 1C). In contrast, the thymus indices were markedly reduced in the same groups, demonstrating substantial thymic involution (Figure 1D), presumably related to chemotherapy (Healthy+CHOP group), tumor-induced immunosuppression (RLS_40_ group), or a combination of both (RLS_40_+CHOP group) (Figure 1D).

Histological analysis of spleens revealed that administration of CHOP to both healthy and RLS_40_-bearing mice significantly affected spleen structure, resulting in the cell depletion of the red and white pulp and a significant reduction in the number and size of lymphoid follicles (Figure 1E, upper panel). Furthermore, administration of CHOP caused red pulp hyperemia with expansion of megakaryocytes and other blood cells, reflecting the toxic effect of polychemotherapy on the animal. It is worth mentioning that the RLS_40_ tumor itself exerted a certain stimulating effect on the spleen, resulting in a slight increase in the number of follicles (Figure 1E, upper panel).

Histological study of thymuses demonstrated a pronounced suppressive effect of CHOP on the immune system of both healthy and tumor-bearing mice. Three consecutive courses of chemotherapy caused significant involution and even atrophy of the thymus, its replacement by connective and adipose tissue, a decrease in the cortex and medulla cellularity, layer inversion, and a massive lymphocyte death (Figure 1E, bottom panel). RLS_40_ tumor growth did not cause such dramatic changes in thymus structure: only a decrease in size and cellularity was observed, while the overall structure of the organ was preserved (Figure 1E, bottom panel).

**Figure 2:**
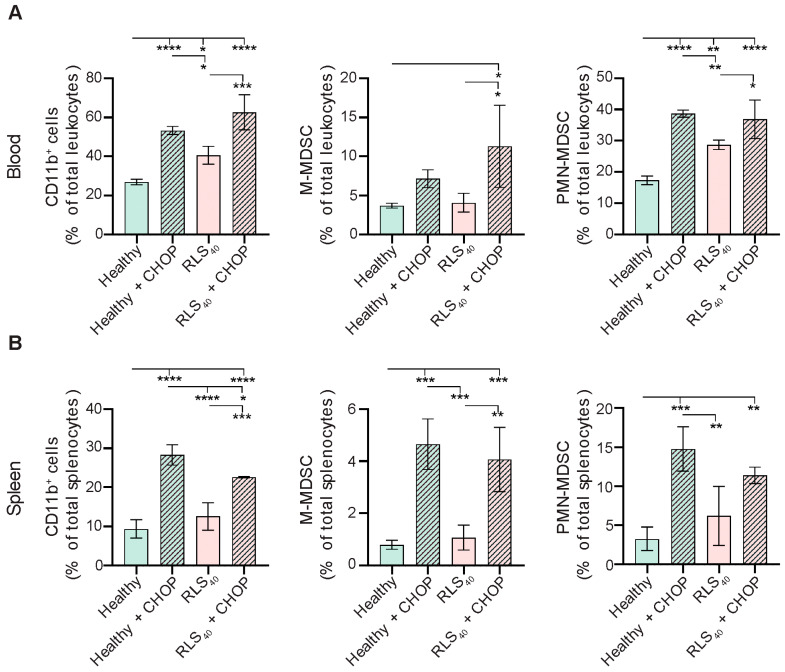
CHOP chemotherapy promotes the accumulation of MDSCs in healthy and RLS_40_-bearing CBA mice. The percentage of CD11b^+^ myeloid cells, CD11b^+^Ly6G^−^Ly6C^high^ M-MDSCs, and CD11b^+^Ly6G^+^Ly6C^low^ PMN-MDSCs in (**A**) peripheral blood and (**B**) spleens of CHOP-treated healthy and RLS_40_-bearing mice was assessed with flow cytometry. Data represent two independent biological experiments (n = 4) and are presented as mean ± SD. Statistical analysis by one-way ANOVA with Tukey’s post hoc test. * *p* < 0.05, ** *p* < 0.01, *** *p* < 0.001, **** *p* < 0.0001.

Immunological consequences of CHOP administration to healthy and RLS_40_-bearing mice were evaluated on day 10 after the third course of CHOP treatment by measuring the total number of myeloid cells (CD11b^+^), and myeloid subpopulations M-MDSCs (CD11b^+^Ly6G^−^Ly6C^high^) and PMN-MDSCs (CD11b^+^Ly6G^+^Ly6C^low^) in blood and spleens of experimental animals (Figure 2) (gating strategy are displayed in Supplementary Materials (Figure S1)).

The obtained data show that the development of RLS_40_ tumor is accompanied by a 1.5-fold increase in CD11b^+^ myeloid cells in the peripheral blood compared to healthy controls (Figure 2A, left panel). PMN-MDSCs constituted the most expanded population, representing up to 28% of total blood leukocytes in tumor-bearing mice (RLS_40_ group) (Figure 2A, right panel). In contrast, the proportion of M-MDSCs remained consistently low (<4%) and comparable to control levels (Figure 2A, central panel). In the spleen, the abundance of myeloid cells (CD11b^+^ cells) was significantly lower relative to peripheral blood in both healthy and tumor-bearing mice (around 1% for M-MDSCs and up to 5% for PMN-MDSCs), and tumor development did not markedly alter this parameter (Figure 2B).

CHOP administration to RLS_40_-bearing mice effectively induced tumor regression, resulting in a 2-fold decrease in tumor weight (Figure S2). Surprisingly, this therapeutic effect of CHOP was accompanied by a pronounced expansion of both PMN-MDSCs (1.2–2 fold) and M-MDSCs (2–4 fold) in the peripheral blood and spleen (Figure 2A,B). Moreover, CHOP administration also induced MDSC expansion in healthy mice, with the levels reaching those observed in CHOP-treated tumor-bearing animals. The most significant increase was observed in the spleen—5-fold (from 1% to 5%) for M-MDSCs and 7.5-fold (from 2% to 15%) for PMN-MDSCs (Figure 2B). Thus, CHOP administration directly induced significant MDSC accumulation in the peripheral blood and, more pronouncedly, in the spleen, irrespective of tumor presence, simultaneously causing involution of the immune organs. This suggests that CHOP may promote systemic immunosuppression, which could potentially limit the therapeutic efficacy of polychemotherapy.

### 2.2. The Effects of CHOP or Its Individual Components on MDSC Expansion Dynamics In Vivo

**Figure 3:**
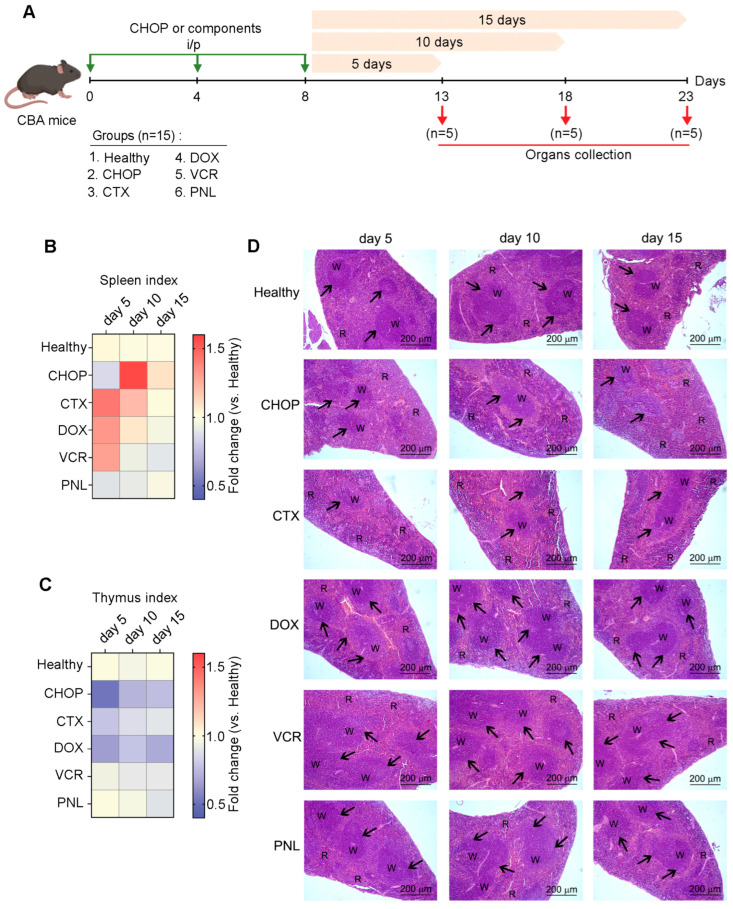
Dynamic changes in indices of immune-related organs induced by either the CHOP regimen or individual CHOP components in healthy CBA mice. (**A**) Experimental setup. Naïve CBA mice received three i/p injections of either CHOP or individual CHOP components according to the experimental schedule. Analysis was performed on days 5, 10, and 15 after the final round of treatment. (**B**,**C**) Heat maps depicting spleen and thymus indices of experimental mice, showing organ-to-body weight ratios normalized to healthy control. Data represent two independent biological experiments (n = 5). (**D**) Representative histological images of the spleen of healthy mice receiving CHOP or its individual components. Hematoxylin and eosin staining. Original magnification ×100. Black arrows indicate lymphoid follicles in the spleen. R: spleen red pulp; W: spleen white pulp.

To investigate the dynamics of MDSCs expansion following CHOP administration and to identify the CHOP component(s) responsible for this immunomodulatory effect, healthy CBA mice received three rounds of either CHOP regimen or individual CHOP components in equivalent doses: CTX (50 mg/kg), DOX (4 mg/kg), VCR (0.1 mg/kg), and PNL (5 mg/kg) (Figure 3A). Subsequently, on days 5, 10, and 15 following the third course of drug administration, five mice from each group were euthanized (see Figure 3A for details). The spleen, thymus, and liver organ indices were measured, and the immune cell profile in the spleen was analyzed, as the spleen was the most affected upon CHOP treatment compared to peripheral blood (Figure 2B). It should be noted that we studied the immunosuppressive effect of CHOP and its components on healthy mice on purpose, because tumors, as previously demonstrated, possess inherent immunomodulatory activity, impacting the structure and function of immune organs.

In the group of mice that received CHOP, the splenic index demonstrated a significant 1.5-fold increase compared to the untreated control on day 10 following the third round of polychemotherapy, demonstrating reactive splenomegaly (Figure 3B). In contrast, individual CHOP components, namely CTX, DOX, and VCR, induced such an effect earlier, with a peak observed on day 5 post-treatment, which subsequently declined by day 10 (Figure 3B). By day 15, splenic index values returned to baseline levels observed in the healthy control across all experimental groups (Figure 3B). Notably, administration of CHOP, CTX, and DOX resulted in a reduced thymic index (Figure 3C), indicating thymic involution and suggesting an adverse effect of cytostatic agents on lymphopoiesis. Interestingly, PNL had a minimal impact on both splenic and thymic indices (Figure 3B,C). Furthermore, hepatic indices remained largely unchanged across all treatment groups, indicating an absence of significant hepatotoxicity for both the CHOP regimen and the individual components (Figure S3).

Histological examination of the spleens of healthy mice after administration of CHOP shows that polychemotherapy caused significant structural changes in the spleen, completely consistent with the picture described earlier (Figure 1E), manifested by significant red pulp hyperemia with an expansion of blood cells, as well as a reduction in lymphoid follicles and their cellular depletion, reflecting both the immunosuppressive effect and the general toxicity of polychemotherapy (Figure 3D). These pathological changes were most pronounced on day 10 after the third course of CHOP, correlating with a marked increase in the splenic index. Similar notable immunosuppressive changes in the spleen were observed on day 5 after CTX administration (Figure 3D), sustaining the significance of this CHOP component in the development of immunosuppression.

When administered as monotherapy, neither DOX nor VCR induced immunosuppressive changes in the spleen. Instead, an increase in the number and size of lymphoid follicles, along with red pulp congestion, was observed (Figure 3D). These alterations peaked on day 5 and gradually decreased, returning to a level comparable to the untreated control by day 15. This temporal pattern correlated with the slight fluctuations in the splenic indices (Figure 3D). PNL administration had no detectable effect on either the splenic index or the spleen’s structural organization (Figure 3D).

A detailed analysis of immune cell profiles in the spleen of healthy mice treated with either CHOP or its components revealed that cytostatic administration induced an expansion of CD11b^+^ myeloid cell subpopulations concurrently with a reduction in T-lymphocyte counts (Figure S4C and S4B, respectively). The most pronounced effects were observed on day 10 following the third round of treatment with CHOP and CTX (Figure S4).

**Figure 4:**
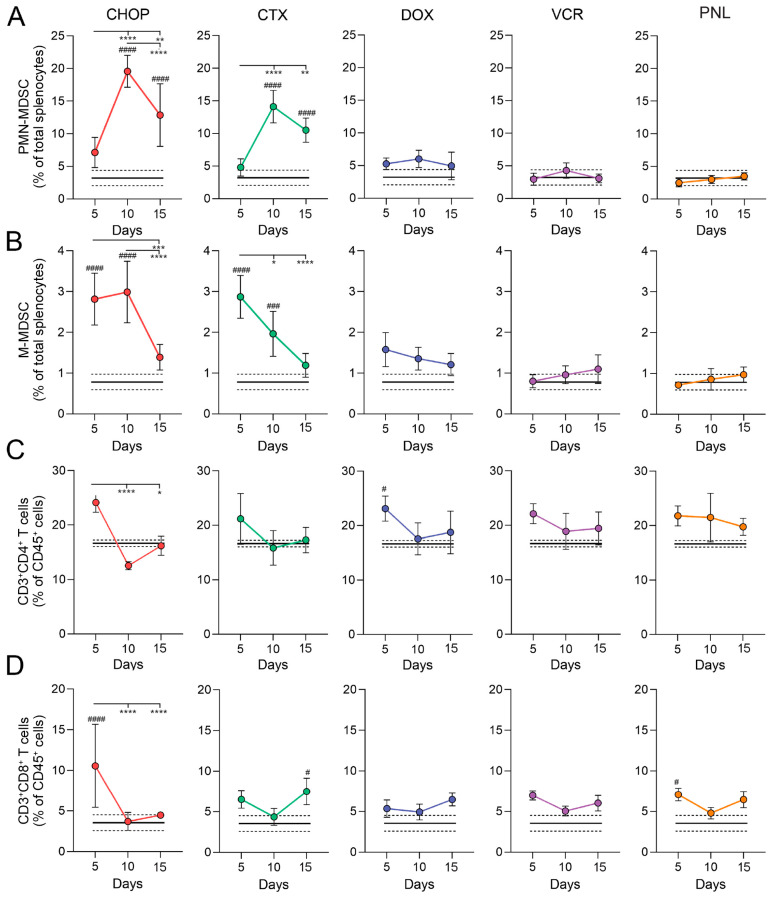
Contribution of individual CHOP components to the splenic immune cell profile in healthy CBA mice. Mice were i/p treated with CHOP polychemotherapy or individual CHOP components (CTX: cyclophosphamide; DOX: doxorubicin; VCR: vincristine; PNL: prednisolone) three times with four-day intervals. Splenocytes were analyzed by flow cytometry at days 5, 10, and 15 after the final round of treatment to assess the frequencies of (**A**) PMN-MDSCs, (**B**) M-MDSCs, (**C**) CD4^+^ T cells, and (**D**) CD8^+^ T cells. Data are presented as mean ± SD (n =5). Black solid and dashed lines represent the mean and SD of untreated control mice (n = 5), respectively. Statistical analysis was performed using two-way ANOVA with Tukey’s multiple comparison tests. # indicates statistically significant differences between the treated group vs. the untreated control. * indicates statistically significant differences within experimental groups across time points. * and # *p* < 0.05, ** *p* < 0.01, *** and ### *p* < 0.001, **** and #### *p* < 0.0001.

Following CHOP treatment, PMN-MDSCs expanded markedly by day 10, reaching ~20% of total splenocytes, while M-MDSCs exhibited a more modest yet statistically significant increase, remaining below 4% (Figure 4A,B). The frequencies of other myeloid populations, including CD11b^+^MHCII^+^ cells, and CD11b^+^F4/80^+^ macrophages, were also increased, though to a lesser extent (<8%) (Figure S4D,E). By day 15, MDSC levels declined but remained elevated compared to the control group (Figure 4A,B). Interestingly, splenic CD4^+^ and CD8^+^ T-cell counts mirrored the expansion of MDSCs: CHOP administration induced an initial transient increase in T cell numbers relative to the untreated control on day 5, which was followed by a sharp decline by day 10 and a subsequent restoration to control levels by day 15 (Figure 4C,D).

Among CHOP components, only CTX administration resulted in immune alterations that closely resembled those in CHOP-treated mice, including a robust expansion of splenic CD11b^+^ cells and PMN-MDSCs by day 10 post-treatment (20% and 15% of total leukocytes, respectively) (Figure 4A and Figure S4C). In contrast, DOX, VCR, and PNL had minimal effects on myeloid cells, including MDSCs (Figure 4 and Figure S4). The T-cell dynamics in the CTX-treated group mirrored MDSC expansion, as it was observed for CHOP treatment: CD4^+^ and CD8^+^ T-lymphocytes transiently increased on day 5, but sharply declined by day 10, coinciding with peak MDSC levels. By day 15, the count of T cells was partially recovered (Figure 4C,D).

**Figure 5:**
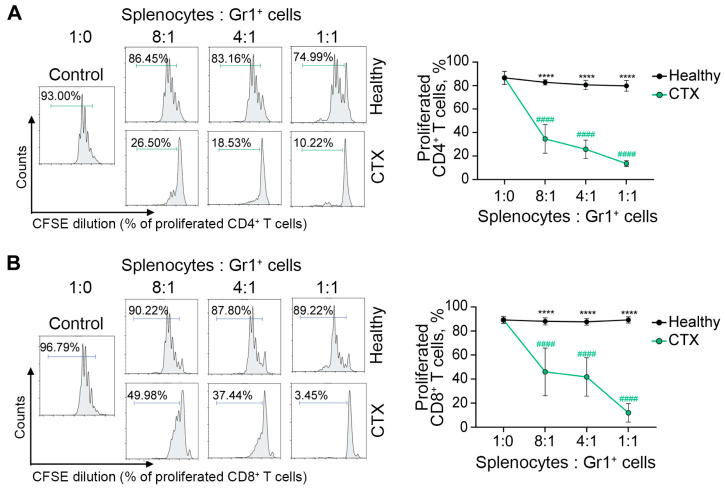
T-cell-suppressive function of CTX-induced MDSCs ex vivo. Splenocytes isolated from healthy mice, labeled with CFSE, and stimulated with ConA were used as responder cells. Responder cells were cultured alone (Control) or either with splenic Gr1^+^ MDSCs isolated from CTX-treated mice (CTX) or splenic Gr1^+^ cells isolated from intact mice (Healthy) at the indicated splenocyte-to-Gr1^+^ cell ratios (8:1, 4:1, and 1:1) for 3 days. Proliferation of (**A**) CD4^+^ and (**B**) CD8^+^ T cells was evaluated by CFSE dilution using flow cytometry. The percentage of proliferating cells is indicated in each histogram gate (left panel) and summarized as mean ± SD (n = 4) (right panel). Statistical analysis was performed using two-way ANOVA with Tukey’s multiple comparison tests. # indicates statistically significant differences compared with control; * indicates statistically significant differences within experimental groups at the same ratio. **** and #### *p* < 0.0001.

To validate the immunosuppressive function of CTX-induced MDSCs, their ability to inhibit T-cell proliferation was evaluated ex vivo. Gr1^+^ MDSCs were isolated from the spleens of CTX-treated mice via positive magnetic sorting, while Gr1^+^ splenic myeloid cells isolated from healthy mice served as the control. The immunosuppressive activity of MDSCs was assessed in an ex vivo co-culture assay with CFSE-labeled, ConA-activated splenocytes (Figure 5). As expected, CTX-induced MDSCs demonstrated potent immunosuppressive activity, significantly suppressing the proliferation of both CD4^+^ and CD8^+^ T cells ex vivo compared to Gr1^+^ control cells (*p* < 0.0001; Figure 5A,B).

Thus, the most pronounced expansion of splenic MDSCs, concomitant with a reduction in lymphoid follicles and T-cell depletion in the spleen, occurred in the mice treated with the CHOP regimen, and CTX largely reproduced these outcomes (Figure 4 and Figure S4). Considering that CTX-induced MDSCs significantly suppressed T-cell proliferation ex vivo, the observed reduction in splenic T-cell numbers in CHOP- and CTX-treated mice (Figure 4) may be directly mediated by their activity. These findings demonstrate that CTX is the principal driver of CHOP-induced immunosuppression, with day 10 representing the peak immunosuppressive phase characterized by MDSC expansion and T-cell depletion.

### 2.3. Modulation of CTX-Induced Immunosuppression by Triterpenoids In Vivo

**Figure 6:**
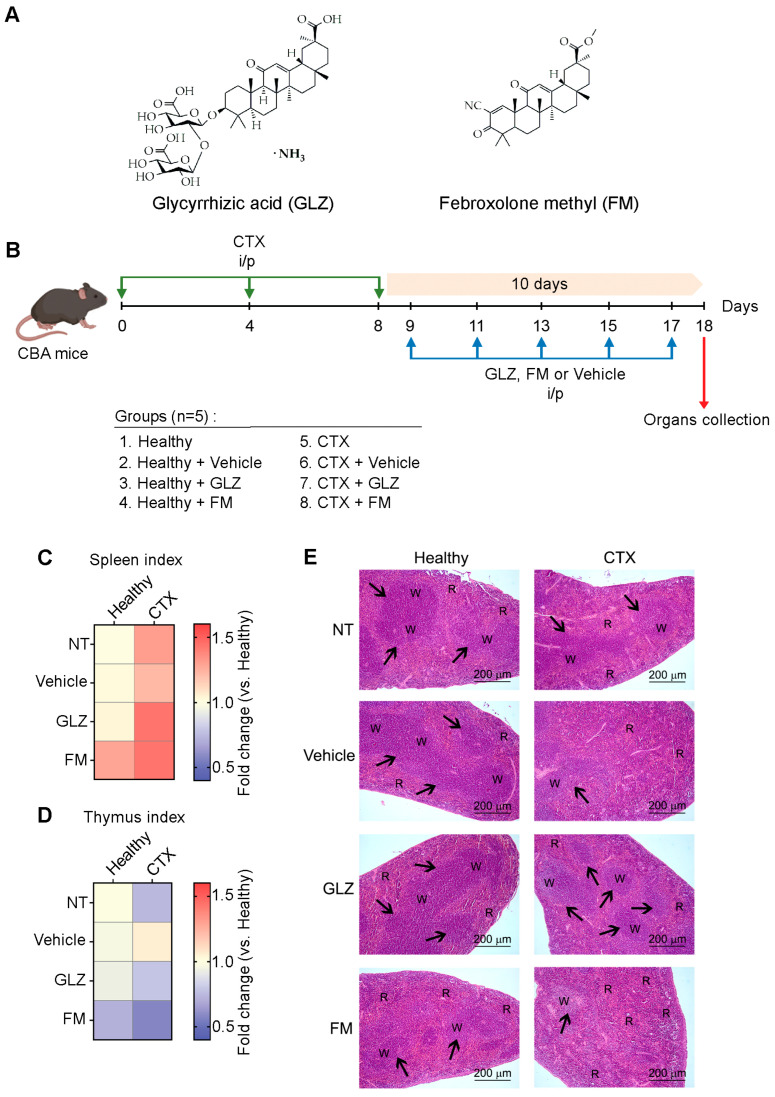
Effects of GLZ and FM triterpenoids on immune organs of healthy and CTX-treated mice. (**A**) Chemical structures of glycyrrhizic acid (GLZ) and febroxolone methyl (FM). (**B**) Experimental setup. Naïve CBA mice received three i/p injections of CTX according to the experimental schedule. The mice were then i/p treated with triterpenoids GLZ or FM (**C**,**D**). Heat maps depicting spleen and thymus indices of experimental mice, showing organ-to-body weight ratios normalized to healthy control. Data represent two independent biological experiments (n = 5). (**E**) Representative histological images of the spleen of healthy and CTX-treated mice after GLZ and FM administration. Hematoxylin and eosin staining. Original magnification ×100. Black arrows indicate lymphoid follicles in the spleen. R: spleen red pulp; W: spleen white pulp.

It is known that natural-based bioactive compounds can be efficiently used to weaken the immunosuppressive activity of MDSCs in vitro and in vivo [43,44,45]. For example, oleanane-type triterpenoids CDDO-Me and GLZ can significantly abrogate the immune suppressive activity of MDSCs by inhibition of STAT3 signaling, reducing the expression of immunosuppressive factors, such as ROS [41], COX-2, PGE2, and Arg-1 [40] in MDSCs, but not affecting the viability of MDSCs or their proportion in the spleens [40,41]. Here, the activity of GLZ and its analog febroxolone methyl (FM), demonstrating structural similarity to CDDO-Me (Figure 6A), was examined in suppressing CTX-induced MDSC accumulation and related immune imbalance in vivo. Healthy CBA mice were treated i/p with CTX according to the CHOP regimen and then received five i/p injections of GLZ, FM, or vehicle (10% Tween-80) with 2-day intervals (Figure 6B). Splenocyte analysis was performed on the day following the final triterpenoid administration, which corresponded to day 10 after the last CTX injection (Figure 6B), when the peak accumulation of MDSCs in the spleen was expected (Figure 4).

Administration of CTX to healthy CBA mice increased the spleen index and reduced the thymus index (Figure 6C,D), without significantly affecting the liver index (Figure S5), compared to healthy controls, similar to previously obtained results (Figure 3B,C and Figure S3). Administration of GLZ or FM to CTX-treated mice led to an additional increase in the spleen indices, indicating that these compounds did not prevent CTX-induced splenomegaly (Figure 6C). Also, both triterpenoid treatments appeared to partially mitigate the CTX-induced thymic involution, with values slightly higher than those of CTX alone (Figure 6D). Notably, FM treatment of CTX-induced mice produced the highest liver index, suggesting a possible mild hepatotoxic effect, while GLZ led to only a minimal increase in liver index in healthy mice (Figure S5A).

Histological examination of the spleens showed that CTX administration caused the immunosuppressive changes described in detail above (decrease in the number and size of lymphoid follicles, decreased cell content of the red and white pulp) (Figure 6E). Administration of the test compounds to healthy mice did not significantly affect the structural organization of the spleen, except for FM, which caused slight red pulp hyperemia (Figure 6E). Administration of GLZ to CTX-induced animals significantly reduced CTX-induced immunosuppression, returning the number and size of splenic lymphoid follicles to healthy levels, while FM administration did not have any positive effect on CTX-induced spleen alterations (Figure 6E).

Histological analysis of the liver revealed that administration of CTX caused certain destructive changes in the liver parenchyma (moderate dystrophy, monocellular and focal necrosis), despite the absence of any noticeable changes in liver indices (Figure S5B). Administration of GLZ abrogates the pathological manifestations of CTX-induced toxicity, returning liver structure to a healthy level. Conversely, administration of FM exacerbated CTX-induced liver destruction (Figure S5B). It should be noted that administration of both GLZ and FM had no damaging effect on the liver of healthy mice (Figure S5A,B).

**Figure 7:**
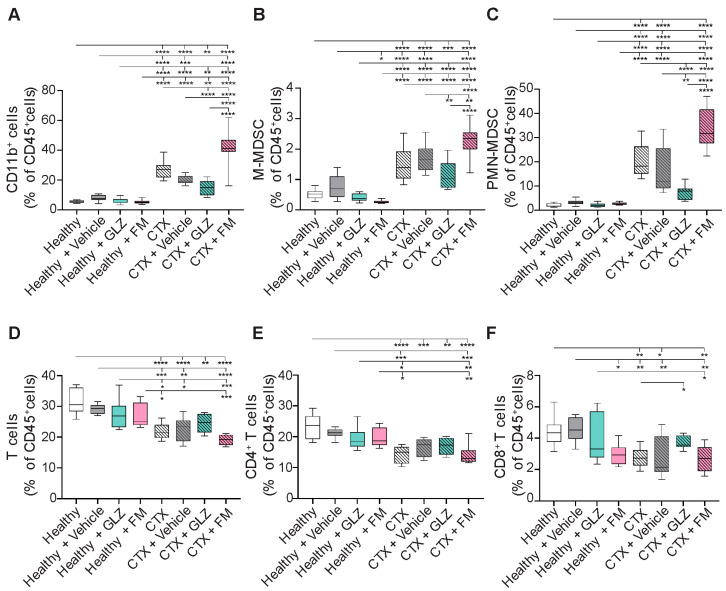
Effects of triterpenoid treatment on redistribution of (**A**) CD11b^+^ cells, (**B**) M-MDSCs, (**C**) PMN-MDSCs, (**D**) total T cells, (**E**) CD4^+^ T cells, and (**F**) CD8^+^ T cells in spleens of healthy and CTX-treated mice (n = 5). Vehicle: 10% Tween-80 in saline. Data are presented as box-and-whiskers plots (median line; whiskers: min to max). Statistical analysis was performed using one-way ANOVA with Tukey’s post hoc test. * *p* < 0.05, ** *p* < 0.01, *** *p* < 0.001, **** *p* < 0.0001.

In healthy mice, neither vehicle nor triterpenoid administration induced significant alterations in both myeloid and T-cell profiles in spleens (Figure 7). CTX treatment was shown to induce accumulation of CD11b^+^ myeloid cells (5-fold, *p* < 0.0001), including both PMN-MDSCs (10-fold, *p* < 0.0001) and M-MDSCs (3-fold, *p* < 0.0001), together with the reduction in CD4^+^ (1.4-fold, *p* < 0.0001) and CD8^+^ T-cell populations (1.6-fold, *p* < 0.01) in the spleens of experimental animals compared to healthy control (Figure 7), which is consistent with our earlier findings (Figure 4). In CTX-treated mice, vehicle injection modestly altered myeloid cell distribution, not-significantly reducing the number of total CD11b^+^ myeloid cells (1.4-fold, *p* = 0.0779) and PMN-MDSCs (1.4-fold, *p* = 0.088) compared to CTX-treated control mice, without significantly affecting T-lymphocyte frequencies (Figure 7A–C). Administration of GLZ to CTX-treated mice significantly reduced splenic accumulation of myeloid cells—CD11b^+^ cells (1.4-fold, *p* < 0.0001), M-MDSCs (1.4-fold, *p* < 0.01), and PMN-MDSCs (2-fold, *p* < 0.0001) (Figure 7A–C)—and tended to increase the abundance of T cells compared to the CTX+Vehicle group (Figure 7D–F), indicating the potential immunoactivating properties of GLZ. In contrast, FM treatment of CTX-induced mice had the opposite effects, further increasing the levels of splenic CD11b^+^ cells (2-fold vs. CTX+Vehicle, *p* < 0.0001; 1.5-fold vs. CTX, *p* < 0.0001), M-MDSCs (1.3-fold vs. CTX+Vehicle, *p* < 0.01; 1.5-fold vs. CTX, *p* < 0.0001), and PMN-MDSCs (2.2-fold vs. CTX+Vehicle, *p* < 0.0001; 1.6-fold vs. CTX, *p* < 0.0001) (Figure 7A–C). At the same time, FM had a minimal effect on T-cell number; the differences from the CTX and CTX+Vehicle groups were not statistically significant (Figure 7D–F).

Thus, we demonstrated that GLZ ameliorates CTX-induced splenic MDSC accumulation and the associated T-cell depletion, as well as structural changes in spleen and liver. Conversely, FM was shown to potentiate the immunosuppressive and toxic effects of CTX, promoting a more pronounced expansion of splenic MDSCs and liver destruction.

### 2.4. The Effects of GLZ and FM on the Characteristics of CTX-Induced MDSCs In Vivo

**Figure 8:**
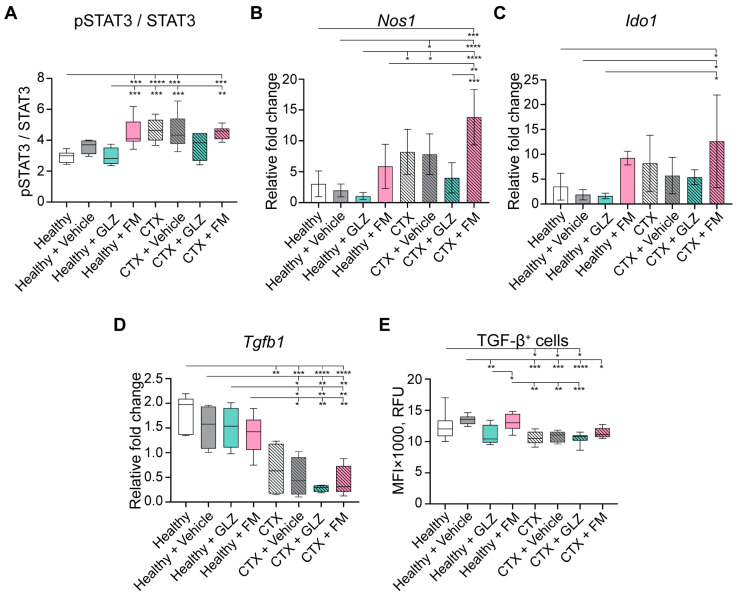
MDCS-related STAT3 signaling and expression of immunosuppressive factors in splenocytes from CTX-treated and healthy mice administered with GLZ and FM. (**A**) Intracellular levels of pSTAT3 and STAT3, presented as the normalized pSTAT3/STAT3 ratio, were assessed by flow cytometry. mRNA expression levels of (**B**) nitric oxide synthase 1 (*Nos1*), (**C**) indoleamine 2,3-dioxygenase 1 (*Ido1*), and (**D**) transforming growth factor β (*Tgfb1*) were quantified by RT-qPCR and normalized to *Hprt* expression. (**E**) Intracellular levels of TGF-β protein were assessed by flow cytometry. The data represent three independent biological experiments. Data are presented as box-and-whiskers plots (median line, whiskers: min to max) (panels **A**,**E**) and as mean ± SD (panels **B**–**D**). Statistical analysis was performed by one-way ANOVA with Tukey’s post hoc test. * *p* < 0.05, ** *p* < 0.01, *** *p* < 0.001, **** *p* < 0.0001.

The activation of the STAT3 signaling pathway promoting expansion and stimulation of the immunosuppressive properties of MDSC [5,44,46,47,48] was studied. The pSTAT3/STAT3 ratio was assessed by flow cytometry in splenocytes from CTX-treated mice that received triterpenoids. It was shown that all experimental groups that received CTX exhibited elevated pSTAT3/STAT3 ratios compared to the intact control mice, confirming STAT3-dependent induction of MDSCs (Figure 8A). Treatment of CTX-induced mice with GLZ or FM did not substantially alter this ratio. Nevertheless, GLZ, but not FM, exhibited some tendency to reduce this ratio to a level comparable to that of the healthy control, although these differences were not statistically significant (Figure 8A). Furthermore, administration of the vehicle to naïve healthy mice induced STAT3 pathway activation, an effect that was markedly amplified by FM, reaching levels similar to those in CTX-treated groups (Figure 8A).

The expression levels of other immunosuppressive genes, *Nos1* and *Ido1*, were elevated in the splenocytes of CTX-treated mice (Figure 8B,C), thereby demonstrating the immunosuppressive potential of CTX-induced MDSCs. GLZ treatment of CTX-administered mice exhibited a tendency to decrease the expression of these genes, thereby potentially reducing the immunosuppressive burden (Figure 8B,C). In contrast, FM resulted in an increase in the expression levels of *Nos1* and *Ido1* (Figure 8B,C). These observations are consistent with the different levels of MDSCs in the spleens of CTX-treated mice receiving GLZ and FM (Figure 7A–C).

Interestingly, splenocytes from CTX-treated mice demonstrated reduced expression of immunosuppressive TGF-β at both mRNA and protein levels compared to those from untreated controls (Figure 8D and Figure 8E, respectively). GLZ administration to mice tended to further reduce TGF-β levels in both the CTX-treated and untreated groups (Figure 8D,E). Undoubtedly, further detailed investigations are required.

In summary, we demonstrated that the in vivo induction of MDSCs with CTX is dependent on STAT3 activation. The elevated levels of splenic MDSCs were found to correlate with augmented *Nos1* and *Ido1* mRNA, along with decreased levels of *Tgfb1* mRNA and TGF-β protein. The administration of GLZ to CTX-treated animals exhibited an immunostimulatory effect, characterized by a reduction in splenic MDSC number, a recovery of T-cell counts, and a tendency to reduce immunosuppressive characteristics of MDSCs. Conversely, FM augmented the immunosuppressive effect of CTX by expanding the splenic population of MDSCs and enhancing their immunosuppressive characteristics.

### 2.5. The Effects of GLZ and FM on the Characteristics of Bone Marrow-Derived MDSCs Generated In Vitro

**Figure 9:**
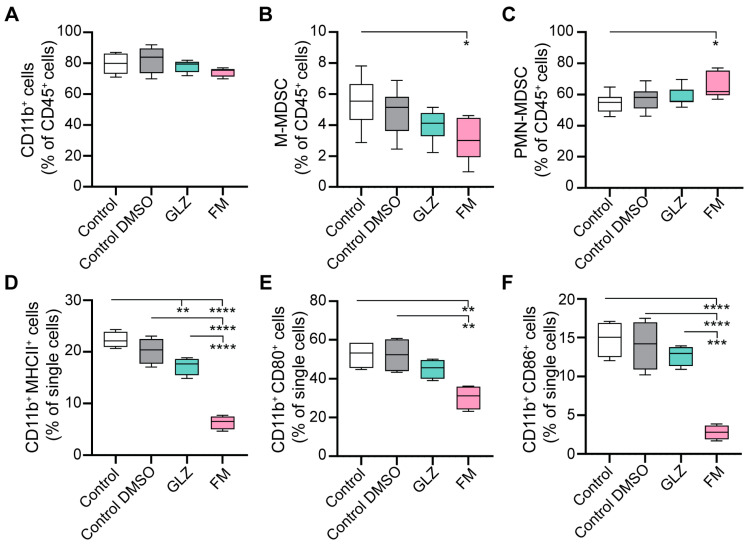
Effects of GLZ and FM on the yield, differentiation, and maturation of BM-MDSCs. BM-MDSCs were generated from bone marrow cells in vitro with GM-CSF in the presence of GLZ or FM. (**A**–**C**) Yield of MDSC populations assessed by flow cytometry: (**A**) total myeloid (CD11b^+^), (**B**) M-MDSCs, and (**C**) PMN-MDSCs. (**D**–**F**) Expression of maturation markers on CD45^+^CD11b^+^ cells assessed by flow cytometry: (**D**) MHC II, (**E**) CD80, and (**F**) CD86. Data are presented as box-and-whisker plots (median line, whiskers: min to max) (n = 4). Statistical analysis was performed using one-way ANOVA with Tukey’s post hoc test. * *p* < 0.05; ** *p* < 0.01; *** *p* < 0.001; **** *p* < 0.0001.

**Figure 10:**
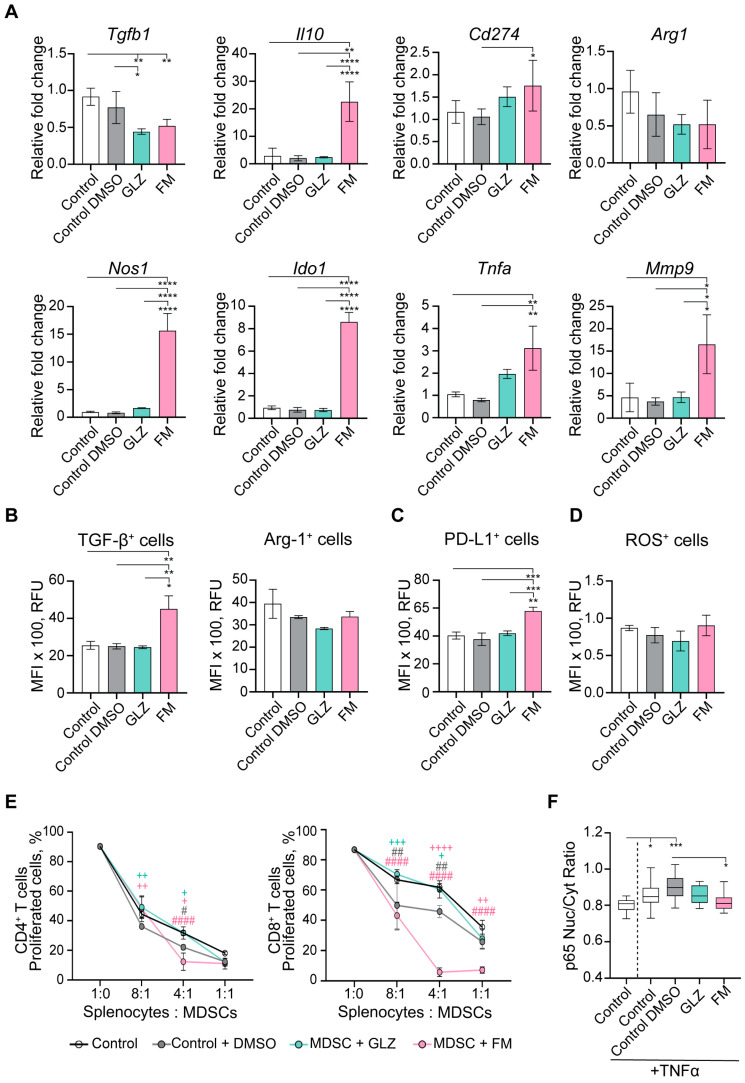
Effects of GLZ and FM on the immunosuppressive profile of BM-derived MDSCs. BM-derived MDSCs were generated in vitro in the presence or absence of GLZ or FM. (**A**) mRNA expression of immunosuppressive genes *Tgfb1*, *Il10*, *Cd274* (*Pd-l1*), *Arg1*, *Nos2*, *Ido1*, *Tnfa*, and *Mmp9* was analyzed by RT-qPCR. (**B**) TGF-β and Arg1 intracellular expression, (**C**) PD-L1 expression, and (**D**) ROS production in CD11b^+^ cells are shown as mean fluorescence intensity (MFI) measured by flow cytometry. (**E**) Proliferation of CFSE-labeled CD4^+^ and CD8^+^ T cells after co-culture with BM-MDSCs for 3 days at splenocyte-to-MDSC ratios of 8:1, 4:1, and 1:1. Data are presented as mean ± SD (two independent experiments, n = 3 per group). (**F**) p65 nuclear translocation in BM-MDSCs was quantified as the ratio of nuclear-to-cytoplasmic p65-specific fluorescence intensity (confocal microscopy data). Statistical analysis for panels (**A**–**D**,**F**) was performed using one-way ANOVA with Tukey’s post hoc test (* *p* < 0.05; ** *p* < 0.01; *** *p* < 0.001; **** *p* < 0.0001), for panel (**E**) using two-way ANOVA with Tukey’s multiple comparisons tests vs. Control (# *p* < 0.05; ## *p* < 0.01; #### *p* < 0.0001) and vs. Control+DMSO (+ *p* < 0.05; ++ *p* < 0.01; +++ *p* < 0.001; ++++ *p* < 0.0001).

To investigate the primary impact of the triterpenoids on MDSC differentiation, maturation state, and immunosuppressive characteristics, we evaluated the effects of GLZ and FM on the in vitro-generated bone marrow (BM)-derived MDSCs (hereinafter, BM-MDSCs). MDSCs were differentiated from BM precursors for four days according to an established protocol [49] in the presence of GLZ or FM. Non-toxic concentrations of the triterpenoids (250 µM GLZ and 250 nM FM) were selected based on the absence of cytotoxicity and no adverse effect on BM-MDSC recovery (Figure S6). The generated BM-MDSCs were then characterized for their phenotype and maturation state (MHC II, CD80, and CD86 expression) (Figure 9), as well as for the expression of immunosuppressive factors, ROS production, NF-κB signaling, and ex vivo immunosuppressive function in inhibiting T-cell proliferation (Figure 10).

GLZ was found to have a minimal effect on the yield of MDSC subpopulations (Figure 9A–C) and slightly reduced their maturation state (Figure 9D–F) during development from BM cells in vitro. Furthermore, GLZ did not alter mRNA levels of key BM-MDSC immunosuppressive factors (except for a reduction in *Tgfb1*) (Figure 10A), the protein levels of TGF-β, Arg1, and PD-L1 (Figure 10B), or ROS production (Figure 10C) in the generated MDSCs.

In contrast, FM affected the BM-MDSC subpopulation ratio by moderately increasing PMN-MDSC yield and decreasing M-MDSC yield (Figure 9B,C). It also markedly impaired BM-MDSC maturation (Figure 9D–F), and significantly upregulated immunosuppressive mRNAs encoding *Il10*, *Nos1*, *Ido1*, *Tnfa*, and *Mmp9* (Figure 10A). Furthermore, FM-treated BM-MDSCs showed increased expression of TGF-β and PD-L1 proteins (Figure 10B). Thus, in contrast to GLZ, FM significantly enhanced the immunosuppressive phenotype of BM-MDSCs generated in vitro.

Functional analysis of the ex vivo-generated MDSCs revealed that BM-MDSCs treated with the 0.05% DMSO vehicle (Control+DMSO group) suppressed the in vitro proliferation of CD4^+^ and CD8^+^ T cells by 20–30% more potently than untreated BM-MDSCs (Control) (Figure 9E). This effect is likely attributable to a non-specific, stress-induced MDSC response to DMSO, even at this low concentration. Treatment of BM-MDSCs with GLZ reduced their immunosuppressive activity, as evidenced by increased proliferation of CD4^+^ and CD8^+^ T cells relative to the Control+DMSO group, restoring T-cell proliferation to levels comparable to those of untreated BM-MDSCs (Figure 9E). Conversely, FM dramatically enhanced MDSC-mediated immunosuppression, resulting in nearly complete inhibition of CD4^+^ and CD8^+^ T-cell proliferation even at a splenocyte-to-MDSC ratio of 4:1 (Figure 9E).

Thus, the obtained in vitro data on the phenotype and functional activity of triterpenoid-treated BM-MDSCs are consistent with the in vivo findings, where GLZ demonstrated modest immunoactivating potential, while FM exhibited strong immunosuppressive effects, driving MDSCs accumulation (with high *Nos1* and *Ido1* expression) and T-cell depletion in the spleens of CTX-treated mice (Figure 7 and Figure 8).

To further investigate the mechanisms by which triterpenoids modulate the immunosuppressive function of MDSCs, we analyzed the NF-κB signaling pathway by assessing the nuclear translocation of the p65 subunit in BM-MDSCs (Figure 10F). Specifically, in vitro-generated BM-MDSCs were pretreated with GLZ or FM and subsequently stimulated with the canonical NF-κB activator TNFα. The cells were then immunostained with p65-specific antibodies and analyzed by confocal microscopy. Activation of the NF-κB pathway was quantified by calculating the nuclear-to-cytoplasmic (Nuc/Cyt) ratio of p65-specific fluorescence intensity (Figure 10F). We confirmed that stimulation of control BM-MDSCs with TNFα induced nuclear translocation of p65 subunit, indicating activation of the NF-κB signaling pathway (Figure 10F). In DMSO-treated BM-MDSCs stimulated with TNFα, p65 nuclear translocation was further elevated relative to the control (Figure 10F), indicating additional non-specific activation of the NF-κB pathway as a cellular response to DMSO. It was demonstrated that triterpenoids, especially FM, had the potential to downregulate NF-κB signaling in BM-MDSCs (Figure 10F). GLZ exhibited a tendency to reduce NF-κB signaling, as evidenced by decreased nuclear p65 localization compared to DMSO-treated, TNFα-stimulated control; however, this reduction was not statistically significant (Figure 10F). In contrast, FM significantly inhibited NF-κB signaling in BM-MDSCs. Compared to the TNFα-stimulated Control+DMSO group, FM treatment significantly reduced p65 nuclear translocation in BM-MDSCs, decreasing it to a level comparable to that of unstimulated control (Figure 10F).

**Figure 11:**
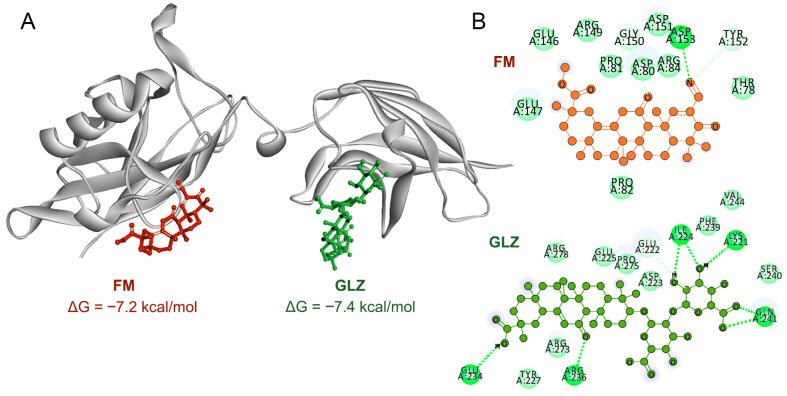
(**A**) Three-dimensional representation of the molecular interactions of FM and GLZ with the DNA-binding domain of p65, generated by blind molecular docking using AutoDock Vina. (**B**) Two-dimensional diagram of the interactions of FM or GLZ with p65. Green dashed lines represent hydrogen bonds.

Blind molecular docking confirmed the ability of both triterpenoids to directly interact with the DNA-binding domain of p65, with comparable Gibbs free energies (Figure 11A). Despite forming a greater number of hydrogen bonds (Figure 11B), the GLZ molecule had a significantly smaller surface contact area with p65 than FM (Figure 11A), which may underlie its less pronounced effect on p65 nuclear translocation.

## 3. Discussion

In tumor-bearing hosts, MDSCs’ expansion is driven by the chronic secretion of tumor-derived myelopoietic and inflammatory mediators. These include colony-stimulating factors (GM-CSF, G-CSF, M-CSF), interleukins (IL-6, IL-10), TGF-β, prostaglandin E_2_ (PGE_2_), S100A8/A9 proteins, and chemokines such as CCL2, CXCL5, and CXCL12. Collectively, these factors induce emergency myelopoiesis and promote the recruitment of immature, immunosuppressive myeloid cells, predominantly PMN-MDSCs, into the peripheral blood and spleen [1,8,12,50]. Indeed, in our RLS_40_ lymphosarcoma model, we observed an approximate 1.5-fold expansion of MDSCs, primarily PMN-MDSCs, in the peripheral blood, whereas no significant accumulation was detected in the spleen.

Unexpectedly, although the CHOP regimen significantly reduced RLS_40_ lymphosarcoma burden (by a 50% in tumor weight), it further increased MDSC accumulation (Figure 2). Furthermore, CHOP induced MDSC expansion in healthy animals to a similar extent as in tumor-bearing mice (Figure 2). Analysis of the individual CHOP components identified CTX as the primary driver of this MDSC accumulation (Figure 4). The capacity of CTX to promote the expansion of MDSCs has been described previously in animal models [39,51,52] and cancer patients [53]. A potential mechanism involves a robust inflammatory response elicited by CTX, which is associated with the release of such factors as GM-CSF, G-CSF, IL-1β, and IL-6, driving myelopoiesis and subsequent MDSC activation and accumulation [54,55,56,57]. Furthermore, T cells were demonstrated to play a critical role in CTX-induced MDSC expansion. In 4T1 tumor-bearing mice, triple CTX administration induced a delayed expansion of MDSCs only in immunocompetent animals. This expansion was driven by an early increase in T-cell activity and IFN-γ production [58]. At the same time, CTX failed to induce MDSC expansion in T-cell-deficient nude mice unless functional T cells were adoptively transferred [58]. In another study, a single high-dose CTX also induced initial activation of CD4^+^ effector cells, which were subsequently tolerized via an MDSC-mediated PD-1/PD-L1 pathway [52]. Notably, we observed a similar initial increase in splenic CD4^+^ and CD8^+^ T-cell numbers on day 5 post-administration of CTX. This was followed by a sharp decline in T-cell numbers by day 10, which coincided with the peak MDSC population. We propose that the initial T-cell activation is a transient, compensatory host response to chemotherapy-induced inflammation and lymphotoxicity. The subsequent drastic reduction in T cells is likely driven by the accumulated CTX-induced MDSCs.

On the one hand, the extent of MDSC expansion is critically dependent on the CTX administration schedule and dosage. For instance, pulsed CTX regimens, which involve high-dose administration either as a single injection or in repeated cycles with short intervals, have been demonstrated to promote MDSC expansion [39,51,52,53]. In contrast, single low dose or metronomic scheduling, characterized by the frequent administration of low doses without extended breaks, does not increase or may even reduce MDSCs and Treg cells, thereby enhancing immune responses in both animal [59,60] and clinical [61] studies. However, metronomic CTX can, in some contexts, lead to an increase in MDSC levels, depending on the specific dose and timing of injections [62,63]. On the other hand, the timing of MDSC analysis post-CTX administration may be even more critical than the dosage and schedule, and could explain some contradictory findings in the literature. In our study, CTX treatment induced a modest increase in splenic MDSCs by day 5, which peaked at day 10 and subsequently declined by day 15, yet remained elevated relative to healthy controls (Figure 4). The aforementioned studies assessed MDSC levels at variable time points following CTX administration, which could lead to conflicting conclusions. For instance, several studies that analyzed MDSC accumulation at early time points (2–3 days post-CTX treatment) concluded that CTX has a minimal effect on MDSC expansion [59]. However, this assessment was likely made before CTX could exert its full effect on MDSCs. While other studies, which quantified MDSCs on days 5–7 post-CTX therapy, documented a moderate increase in splenic levels [36,39], a more significant expansion might have been detected with a delayed measurement. Other reports are consistent with our data, observing a peak of MDSC expansion around day 10 post-CTX (days 9–12) [51,54,64], followed by a return to baseline levels by days 15–20 [51]. Collectively, these findings suggest that the kinetics of MDSC accumulation following CTX administration may be, to some extent, a universal phenomenon, independent of the specific CTX treatment regimen or tumor-bearing status of the experimental animals. We propose that this process is initiated by the cytotoxic effect of CTX on rapidly dividing BM progenitor cells. This effect triggers transient leukopenia, leading to a reduction or even a transient absence of MDSCs during the early post-treatment phase (the first 2–3 days post-CTX). Around days 5–7, emergency myelopoiesis is activated in response to tissue damage, cell death, and the release of DAMPs and pro-inflammatory cytokines. These signals drive hematopoietic progenitors to repopulate myeloid lineages, favoring the differentiation of immature and immunosuppressive MDSCs. Between days 7 and 12, MDSCs accumulate in the peripheral blood and colonize the spleen, reaching the peak of their expansion. Subsequently, by approximately days 15–20 post-CTX treatment, steady-state hematopoiesis is restored and inflammatory signaling subsides. These changes promote the immune-mediated clearance of MDSCs or their differentiation into mature myeloid cells, ultimately resulting in a gradual return to baseline levels by day 20. It should be mentioned that the observed kinetics of CTX-induced MDSC expansion in mice may, to some extent, be recapitulated in patients, although this requires further investigation.

It is worth noting that CTX-induced MDSCs can represent a convenient model for investigating MDSC-mediated immunosuppression, due to the ease of their in vivo induction by CTX. Although CTX-induced MDSCs differ from their tumor-associated counterparts, most notably in their lower immunosuppressive activity [39], ex vivo incubation with IFN-γ moderately aligns their functional profiles [39]. Furthermore, our results significantly differ from those reported by Mikyšková R. et al. [39]. In their study, CTX-induced MDSCs only modestly reduced CD4^+^ T-cell proliferation by 18% compared to the control, an effect that improved to 52% after IFN-γ activation. The suppression of CD8^+^ T-cell proliferation was even weaker, reaching only 24% inhibition post-activation. In contrast, in our work, CTX-induced MDSCs strongly suppressed both CD4^+^ and CD8+ T-cell proliferation by ~85% at a 1:1 splenocyte-to-MDSC ratio, even without IFN-γ activation. These differences in suppressive activity of CTX-induced MDSCs can be explained primarily by distinct experimental conditions, including the use of different mouse strains, CTX dosages, and administration regimens (a single high dose [39] vs. three injections of lower doses in our study). Timing of cell harvesting (7 d [39] vs. 10 d post-CTX treatment) may also contribute to the enhanced suppressive function of MDSCs observed in our settings.

Notably, other components of the CHOP regimen, namely DOX and PNL, did not induce MDSC expansion at the dosages and schedule we employed. This finding contrasts with previous reports where DOX was shown to selectively deplete MDSCs and restore T- and NK-cell populations [65] or, conversely, to promote MDSC activation and expansion under specific experimental conditions [53,66]. Glucocorticoids have been shown to promote MDSC differentiation and function in inflammatory settings through glucocorticoid receptor signaling [67,68]. These divergent results demonstrate that the specific chemotherapeutic combination and administration protocol critically shape the immunological outcome.

To mitigate CTX-induced expansion of immunosuppressive MDSCs, we proposed a strategy of combining chemotherapy with triterpenoids. This class of compounds modulates immune responses and counteracts tumor-induced immunosuppression. Their activity arises from targeting key pathways, including NRF2 antioxidant signaling, NF-κB/MAPK, and JAK/STAT, and inhibiting immunosuppressive enzymes like COX-2, Arg-1, and iNOS [40,69,70]. Indeed, combining the triterpenoid ursolic acid with polychemotherapy [71] or CDDO-Me with the anticarcinogenic rexinoid LG100268 [72] demonstrated synergistic anti-tumor efficacy and substantially suppressed tumor growth. Based on this, we selected the natural triterpenoid GLZ and a novel semisynthetic cyano enone-bearing analog, febroxolone methyl (FM), as candidate agents to ameliorate CTX-induced MDSC accumulation in vivo.

The structural differences between these two molecules can underlie their biological effects. GLZ carries a bulky diglucuronide moiety that reduces electrophilic reactivity and favors selective protein interactions. Specifically, GLZ binds directly to high mobility group box 1 (HMGB1) [73], a nuclear protein that functions as a damage-associated molecular pattern (DAMP) and promotes inflammation via TLR4 and RAGE signaling [74]. By sequestering HMGB1, GLZ inhibits TLR4-mediated activation of NF-κB and MAPK pathways [75,76], thereby attenuating pro-inflammatory responses. GLZ also blocks AKT/mTOR/STAT3 signaling [40,77], a pathway that is essential for MDSCs. We demonstrated that GLZ treatment showed a tendency to downregulate STAT3 and NF-κB signaling pathways in MDSCs, resulting in reduced splenic MDSC numbers, downregulated *Nos1* and *Ido1* expression, partial restoration of T-cell populations in vivo, and reduced MDSC-mediated suppression of T-cell proliferation ex vivo. These results align with published data demonstrating that GLZ inhibits pSTAT3 signaling, downregulates COX-2, PGE_2_, and Arg-1, and suppresses MDSC- and Treg-mediated immunosuppression [40,77].

In the case of FM, we also anticipated a reduction in CTX-induced immunosuppression in mice, as its closest structural analog, CDDO-Me, has demonstrated pronounced immunomodulatory potency both in vitro and in vivo. The presence of a cyano enone pharmacophore in the structure enables CDDO-Me and related compounds to interact directly with various signaling hubs crucial for MDSCs activation, such as components of the JAK/STAT3 and NF-κB pathways [1,78]. For instance, CDDO-Me was shown to effectively abrogate the suppressive activity of splenic MDSCs from EL-4 thymoma-bearing mice against antigen-specific CD8^+^ T cells, primarily by mitigating oxidative stress [41]. Furthermore, in combination with gemcitabine, it significantly enhanced T-cell responses in patients with pancreatic cancer [41]. Surprisingly, the administration of FM following CTX treatment produced the opposite effect, resulting in a pronounced immunosuppressive state (Figure 6C,D, Figure 7 and Figure 8B,C). FM administration significantly amplified CTX-driven splenic MDSC accumulation (Figure 7A–C), which was accompanied by the up-regulation of *Nos1* and *Ido1* (Figure 8B,C), and failure to restore T-cell frequencies (Figure 7D–F). Subsequent in vitro assays revealed that FM promotes the differentiation of BM progenitors into highly immature PMN-MDSCs with enhanced immunosuppressive properties (Figure 9 and Figure 10). Examination of two principal signaling pathways responsible for activating and enhancing MDSC suppressive activity revealed that FM not only failed to activate STAT3 in CTX-induced MDSCs in vivo (Figure 8A), but also downregulated NF-κB signaling in BM-MDSCs (Figure 10F). Therefore, the significant enhancement of MDSC immunosuppressive function by FM appears to be mediated through alternative signaling pathways, which require further investigation.

Despite these discrepancies, our results are consistent with the data reported by Wei H.-J. et al., who described the immunosuppressive activity of another cyano enone-bearing triterpenoid, CDDO-DFPA, on tolerogenic dendritic cells (DCs) [79]. Similarly to our finding, CDDO-DFPA was found to upregulate the expression of TGF-β and IL-10 in DCs, resulting in the inhibition of T-cell responses in an experimental autoimmune encephalomyelitis (EAE) model [79]. Thus, despite sharing the same pharmacophore (the cyano enone moiety), FM (CDDO-DFPA) and CDDO-Me exert opposing effects on the immunosuppressive state. This divergence may be attributed to structural differences in their C and E rings. Consistent with this hypothesis, our data show no effect of FM on STAT3 phosphorylation in contrast with CDDO-Me, which effectively suppresses STAT3 phosphorylation by direct binding to Cys^259^ [78,80]. Given that MDSC expansion can depend not only on STAT3 but also on NF-κB signaling [81], and considering that oleanane-type triterpenoids, including SM, can block this pathway [42], we propose that the observed effects of FM are linked to this mechanism. However, this hypothesis requires further experimental validation in future work.

Thus, our study provides important insights into the immunosuppressive action of CHOP chemotherapy, confirming the in vivo induction of MDSCs by CTX. We have systematically characterized the dynamics of MDSC accumulation and T-cell suppression, defined the functional properties of CTX-induced MDSCs, and explored the potential of triterpenoids to correct this CTX-induced immunosuppression. Our findings could help researchers in efforts to enhance the efficacy of antitumor chemotherapy and develop novel strategies for mitigating MDSC-mediated immunosuppression.

### Limitation of the Study

The main limitation of the study is the partial characterization of the intracellular signaling pathways mediating immunomodulatory effects of triterpenoids on CTX-induced MDSCs. While the STAT3 and NF-κB pathways were examined, further detailed investigation of other signaling pathways is necessary.

## 4. Materials and Methods

### 4.1. Reagents and Equipment

Reagents: RPMI 1640 medium (Thermo Fisher Scientific, #27016-021, Waltham, MA, USA), fetal bovine serum (FBS) (Dia-M, #FSA100SA, Moscow, Russia), bovine serum albumin (BSA) (Dia-M, #BSA.0025, Moscow, Russia), antibiotic/antimycotic solution (MP Biomedicals, #091674049, Santa Ana, CA, USA), gentamicin (Sigma-Aldrich, #G1397-10mL, St. Louis, MO, USA), Ficoll-paque PREMIUM 1.084 g/mL (Cytiva, #17544602, Marlborough, MA, USA), 2-mercaptoethanol (Sigma-Aldrich, #M6250, St. Louis, MO, USA), HEPES (Sigma-Aldrich, #H0887, St. Louis, MO, USA), GlutaMAX Supplement (Gibco, #35050061, Grand Island, NY, USA), rmGM-CSF (Sigma-Aldrich, #G0282, St. Louis, MO, USA), trypan blue (Bio-Rad, #1450021, Hercules, CA, USA), Tween-20 (Sigma-Aldrich, #P1379, St. Louis, MO, USA), TRIzol Reagent (Invitrogen, Ambion, #15596018, Austin, TX, USA), RT buffer (Biolabmix, #R03-008-20, Novosibirsk, Russia), M-MuLV-RH revertase (Biolabmix, #R03-008-20, Novosibirsk, Russia), BioMaster HS-qPCR SYBR Blue (2×) (Biolabmix, #MHC030-007-22, Novosibirsk, Russia), Dynabeads™ Sheep anti-Rat IgG (Thermo Fisher Scientific, Waltham, MA, USA), carboxyfluorescein succinimidyl ester (CFSE) (Invitrogen, #C34554, Austin, TX, USA), 2,7′-dichlorodihydrofluorescein diacetate (DCF-DA) (Sigma-Aldrich, #287810, Darmstadt, Germany), poly-L-lysine solution (Sigma-Aldrich, Darmstadt, Germany), DAPI (Thermo Fisher Scientific, #62248, Austin, TX, USA), fluoromount-G mounting medium (Invitrogen, Thermo Fisher Scientific, #00-4958-02, Austin, TX, USA).

Monoclonal anti-mouse antibodies: anti-CD16/CD32 (BD Biosciences, #553142, clone 2.4G2, San Diego, CA, USA), CD45-FITC (BD Biosciences, #553080, clone 30-F11, San Diego, CA, USA), CD11b-PerCP (Elabscience, #E-AB-F1081UF, clone M1/70, Houston, TX, USA), Ly6C-BV605 (Sony, #1240180, clone HK1.4, San Jose, CA, USA), Ly6G-PE (Elabscience, #E-AB-F1108UD, clone 1A8, Houston, TX, USA), MHCII-FITC (Elabscience, #E-AB-F0990UC, clone M5/114, Houston, TX, USA), F4/80-PB (Sony, #1215615, clone BM8, San Jose, CA, USA), CD3-PE-Cy7 (Elabscience, #E-AB-F1013H, clone 17A2, Houston, TX, USA), CD4-PE (Elabscience, #E-AB-F1097D, clone GK1.5, Houston, TX, USA), CD8-PerCP (Elabscience, #E-AB-F1104F, clone 53-6.7, Houston, TX, USA), CD25-APC (Elabscience, #E-AB-F1102E, clone PC-61.5.3, Houston, TX, USA), Ly-6G/Ly-6C (Gr-1) (Elabscience, Ca#E-AB-F1120A, clone RB6-8C5, Houston, TX, USA), PD-L1-APC (Elabscience, #E-AB-F1132E, clone 10F.9G2, Houston, TX, USA).

Polyclonal anti-mouse antibodies: anti-IgG-AF488 (Abcam, #ab150077, Cambridge, UK), TGF-β (Affinity Biosciences, #AF1027, Hong Kong, China), Arg-1 (Affinity Biosciences, # DF6657, Hong Kong, China), phospho-STAT3 (ABclonal, #AP0070, Wuhan, China), STAT3 (ABclonal, #A1192, Wuhan, China), NF-kB p65/RelA (ABclonal, # A19653, Wuhan, China).

### 4.2. Triterpenoids

GLZ was obtained from JSC Chimpharm (Shymkent, Kazakhstan). FM was synthesized and characterized as described in refs. [82,83]. For the in vitro experiments, stock solutions of GLZ and FM were prepared by dissolving the compounds in DMSO at 0.5 M (GLZ) and 0.5 mM (FM), and were kept at −20 °C until use. For the in vivo experiments, solutions of GLZ and FM were freshly prepared before each injection. Briefly, GLZ or FM compounds were dissolved in DMSO and mixed with Tween-80 (10% of the final volume). The vehicle control solution consisted of DMSO mixed with Tween-80 (10% of the final volume). DMSO was removed under vacuum using an Eppendorf Concentrator 5301 (Eppendorf, Jena, Germany). The resulting films containing triterpenoids and Tween-80 were reconstituted in saline to a final concentration of 250 mg/mL of triterpenoids. An equal volume of saline was added to the vehicle control. The mixtures were vigorously shaken with a vortex for 5 min. The resulting GLZ or FM solution was administered intraperitoneally (i/p) to mice at a dose of 50 mg/kg in a 200 µL volume (n = 5), a dose selected based on prior published work demonstrating the efficacy of oleanane-type triterpenoids with minimal toxicity in mice [84]. Mice in the vehicle control group (n = 5) received i/p injection of 10% Tween-80 in saline in a 200 µL volume.

### 4.3. Mice

10–14-week-old male CBA/LacSto (hereinafter, CBA) were obtained from the vivarium of ICBFM SB RAS (Novosibirsk, Russia). Mice were housed in plastic cages in standard daylight conditions. Water and food were supplied ad libitum. All animal procedures were conducted in strict compliance with the guidelines for the proper use and care of laboratory animals (ECC Directive 2010/63/EU) and ARRIVE guidelines 2.0 [85,86]. The experimental protocols were approved by the Committee on the Ethics of Animal Experiments with the Institute of Cytology and Genetics SB RAS (ethical approval number 188 from 3 October 2024).

### 4.4. Tumor Model

The murine drug-resistant lymphosarcoma RLS_40_ model was utilized. This model was originally developed in our laboratory from chemotherapy-sensitive lymphosarcoma LS [87]. The RLS_40_ cells were maintained in the complete IMDM medium supplemented with 10% FBS and 1% antibiotic-antimycotic solution in the presence of 40 nM vinblastine in a humidified atmosphere containing 5% CO_2_ at 37 °C and were regularly passaged to maintain exponential growth. Solid tumors were induced in mice by intramuscular (i/m) injection of RLS_40_ cells (10^6^ cells/mouse suspended in 100 µL of PBS) into the right thighs of the animals. Tumor-bearing mice were sacrificed under isoflurane anesthesia on day 23 after the tumor transplantation. Tumor weight was measured by subtracting the weight of the intact contralateral leg from the weight of the tumor-bearing leg.

### 4.5. Animal Experiments

#### 4.5.1. The Effects of CHOP Polychemotherapy on MDSC Expansion in RLS_40_-Bearing and Healthy Mice

Healthy and RLS_40_-bearing CBA mice (see Section 4.4 for details) received three courses of CHOP polychemotherapy starting on the fifth day of tumor growth in the case of tumor-bearing animals. Each course consisted of subsequent i/p injections of CTX (50 mg/kg), DOX (4 mg/kg), VCR (0.1 mg/kg) at doses corresponding to one-fifth of their LD_50_, along with PNL (5 mg/kg). Courses were administered at 4-day intervals. Control groups of healthy and RLS_40_-bearing mice received i/p injections of saline. On day 10 after the third course of CHOP (day 23 post-tumor transplantation), mice were euthanized via cervical dislocation under isoflurane anesthesia. Blood, spleen, and other organs (thymus, liver, and kidney) were collected for subsequent analysis.

#### 4.5.2. The Immunomodulatory Effects of CHOP Components on Immune Cell Landscape in Healthy Mice

Healthy CBA mice were treated with the CHOP polychemotherapy regimen or its components CTX (50 mg/kg), DOX (4 mg/kg), VCR (0.1 mg/kg), or PNL (5 mg/kg) administered separately according to the CHOP schedule described above. Mice were euthanized on days 5, 10, and 15 following the third course of CHOP or its individual components (n = 5 from each group per time point). The spleen and other organs (thymus, liver, and kidney) were collected for subsequent analysis.

#### 4.5.3. The Effects of Triterpenoids on CTX-Induced Expansion of MDSC

Healthy CBA mice received three i/p injections of CTX (50 mg/kg) at 4-day intervals. Starting the next day after the third course of CTX administration, mice received five i/p injections of ehicle (10% Tween-80 in saline), GLZ, or FM (50 mg/kg in 10% Tween-80 in saline) at 2-day intervals. Before each injection, GLZ and FM solutions were freshly prepared from stock solutions (see Section 4.2 for details). Mice were euthanized 24 h after the fifth triterpenoid injection, which corresponded to day 10 following the third course of CTX. The spleen and other organs (thymus, liver, and kidney) were then isolated for subsequent analysis.

### 4.6. Isolation of Peripheral Blood Monocytes

Peripheral blood was collected from the retro-orbital sinus of the mice using EDTA-coated tubes to prevent blood coagulation. Red blood cells were lysed with RBC lysis buffer (0.15 M NH_4_Cl, 10 mM NaHCO_3_, and 0.1 mM EDTA) for 5 min at room temperature, followed by washing with PBS buffer. The viability of isolated blood cells was evaluated by the trypan blue exclusion assay. Blood cells were resuspended in staining buffer (PBS supplemented with 2% FBS) for subsequent flow cytometry analysis.

### 4.7. Splenocyte Isolation

Excised spleens were placed in a sterile Petri dish containing 5 mL PBS and gently homogenized using the sterile syringe plunger. The cell suspension was passed through a 70 μm cell strainer (Corning, Glendale, AZ, USA) to obtain a single-cell suspension. Red blood cells were lysed with RBC lysis buffer, followed by washing with PBS. Cell viability was measured by the trypan blue exclusion assay.

### 4.8. Histological Analysis

Before histological analysis, the organs studied were weighed, and organ indices were calculated as (organ weight/body weight) × 100%, followed by normalization to healthy mice.

The spleen and other organs (thymus, liver, and kidney) were collected and fixed in 10% neutral-buffered formalin (BioVitrum, Moscow, Russia), dehydrated in ascending ethanols, and embedded in HISTOMIX paraffin (BioVitrum, Russia). Paraffin sections (up to 5 μm) were sliced on a Microm HM 355S microtome (Thermo Fisher Scientific, Waltham, MA, USA) and stained with hematoxylin and eosin. Images were examined and scanned using an Axiostar Plus microscope equipped with Axiocam MRc5 digital camera (Zeiss, Oberkochen, Germany) at magnification ×100 and ×400.

### 4.9. Positive Selection of Gr 1^+^ Cells

Gr-1^+^ cells were isolated from splenocytes (see Section 4.7 for details) obtained from either healthy or CTX-treated mice (see Section 4.5.2 for treatment details). The isolation was performed using rat IgG anti-mouse Ly-6G/Ly-6C (Gr1) and Dynabeads™ Sheep anti-Rat IgG according to the manufacturer’s instructions, with some modifications as described in [88]. The viability of the positively selected Gr-1^+^ cells was evaluated using the trypan blue exclusion assay. Cell yield and viability were determined in a Goryaev counting chamber under an Axiostar Plus microscope (Zeiss, Munich, Germany).

### 4.10. T-Cell Proliferation Assay

The spleens were isolated from CBA mice and homogenized in PBS using a sterile syringe plunger. The splenocytes were filtered through a 70 µm cell strainer, and lymphocyte-enriched splenocytes were then isolated from splenocytes by density gradient centrifugation on Ficoll-paque PREMIUM 1.084 g/mL at 450× *g*, 22 °C for 30 min. The cells were washed twice with PBS and labeled with 2.5 µM carboxyfluorescein succinimidyl ester (CFSE) (Invitrogen, Cat# C34554) in PBS at 37 °C for 15 min. Following labeling, the CFSE-labeled splenocytes were washed twice with complete RPMI-1640 medium and co-cultured with isolated Gr-1^+^ intact cells, Gr1^+^ CTX-induced MDSCs, or BM-derived MDSCs in 96-well plates.

CFSE-labeled lymphocyte-enriched splenocytes (5 × 10^5^ cells per well) were seeded with isolated Gr1^+^ cells or BM-MDSCs (ranging from 0.625 × 10^5^ to 5 × 10^5^ cells per well) in complete RPMI medium supplemented with 5 ng/mL recombinant mouse IL-2 and 1 µg/mL concanavalin A. The co-cultures were incubated for 72 h under standard conditions (37 °C, 5% CO_2_).

After incubation, cells were stained with anti-mouse CD3-PE-Cy7, CD4-PE, and CD8-PerCP antibodies. CFSE fluorescence of T cells was analyzed using a NovoCyte 3000 flow cytometer (ACEA Biosciences, San Diego, CA, USA). The percentage of proliferating T cells was determined by quantifying CFSE dilution compared to non-activated control T cells.

### 4.11. Evaluation of Immunomodulatory Effects of Triterpenoids on In Vitro Generated MDSCs

#### 4.11.1. Generation of BM-Derived MDSCs

Generation of BM-derived MDSCs was performed according to previously published work [49]. Briefly, healthy CBA mice were anesthetized with isoflurane and euthanized by cervical dislocation. BM cells were flushed from the femurs and tibias of mice with sterile PBS, followed by density gradient centrifugation on Ficoll-paque PREMIUM 1.084 g/mL. Resulting BM cells were pooled from ten CBA mice and cryopreserved. To generate MDSCs, the BM cells were defrosted and cultured in 6-well plates for 4 days in complete RPMI medium (containing 10% FBS, 2% antibiotic/antimycotic solution, 50 µg/mL gentamicin, 50 mM 2-mercaptoethanol, 20 mM HEPES, 20 mM L-glutamine), supplemented with recombinant murine GM-CSF (50 ng/mL). On day 2, the medium was replaced with fresh complete medium supplemented with cytokines.

#### 4.11.2. Cell Viability Analysis

To select the optimal concentrations of GLZ and FM triterpenoids for in vitro experiments, the cytotoxicity of GLZ and FM was evaluated with respect to BM-derived MDSCs using the WST-1 test. BM cells were seeded in 96-well plates at 5 × 10^4^ cells/well in complete RPMI medium supplemented with GM-CSF and triterpenoids (GLZ at 1 µM to 2 mM, FM at 15 nM to 8 µM) or corresponding concentrations of DMSO (up to 0.4%) (vehicle control). Cells were incubated for 4 days under standard conditions. On day 4, WST-1 reagent was added according to the manufacturer’s protocol. Absorbance readings were taken at 570 nm (test wavelength) and 620 nm (reference wavelength) using a Multiscan RC plate reader (Labsystems, Vantaa, Finland). Viability was expressed relative to vehicle-treated control cells; the untreated control was set as 100%.

#### 4.11.3. Immunomodulatory Potential of Triterpenoids on In Vitro Generated MDSCs

BM-derived MDSCs were generated as described above, in the presence of either GLZ (250 µM) or FM (250 nM). On day 2, the medium was replaced with fresh complete medium supplemented with cytokines and the respective triterpenoids. On day 4, prior to analysis, BM-MDSCs were detached using a cell scraper and resuspended in fresh, appropriate culture medium. The cells were pelleted by centrifugation at 400× *g*, 25 °C for 7 min. The supernatant was discarded, and the cells were resuspended in PBS, staining buffer, or culture medium for further analysis.

### 4.12. Flow Cytometry Analysis

The phenotype of immune cells was assessed by the expression of extracellular and intracellular markers using flow cytometry. A total of 1 × 10^6^ of peripheral blood cells or splenocytes in 100 µL of staining buffer (PBS supplemented with 2% FBS) were used in every test. Firstly, cells were Fc-blocked with anti-mouse CD16/CD32 IgG antibodies according to the manufacturer’s recommendations. To characterize the immune cells, the anti-mouse monoclonal antibodies were used, including anti-CD45-FITC, CD11b-PerCP, Ly6C-BV605, Ly6G-PE, F4/80-PB, MHCII-FITC, PD-L1-APC, CD3-PE-Cy7, CD4-PE, CD8-PerCP, CD25-APC, and anti-mouse polyclonal antibodies anti-phospho-STAT3, STAT3, TGF-β, and Arg-1. For extracellular staining, the cells were stained with antibodies (1:100) for 30 min at room temperature, washed twice with staining buffer, and then fixed in 2% formaldehyde in PBS. For intracellular staining, the cells were fixed in 2% formaldehyde in PBS for 15 min at room temperature. Then, cells were permeabilized with permeabilization buffer (0.1% Tween-20, 0.5% BSA in PBS) for 15 min at room temperature and blocked with blocking buffer (0.05% Tween-20, 1% BSA in PBS) for 30 min at room temperature. The permeabilized and blocked cells were stained with primary antibodies (1:100) for 30 min at room temperature, followed by staining with secondary polyclonal anti-mouse IgG-AF488 antibodies (1:1000) for 30 min at room temperature in the dark. The cells were washed twice with staining buffer after each step, and finally, the cells were resuspended in staining buffer. Flow cytometry measurements were performed using a NovoCyte 3000 flow cytometer (ACEA Biosciences, San Diego, CA, USA), and at least 10,000 events were acquired for each sample. The data were processed using NovoExpress software v. 1.1.0 (ACEA Biosciences, San Diego, CA, USA). Gating strategies for MDSCs are shown in Figure S1.

### 4.13. ROS Detection

The oxidation-sensitive dye 2,7′-dichlorodihydrofluorescein diacetate (DCF-DA) was used to measure ROS production by cytokine-induced MDSCs. MDSCs (0.5 × 10^6^) were incubated at 37 °C in a PBS buffer in the presence of 10 µM DCF-DA for 30 min in the dark. Cells were then washed twice with cold PBS and labeled with anti-CD11b-PerCP, anti-Ly6C-BV605, and anti-Ly6G-PE antibodies on ice for 1 h. The cells were washed twice with cold PBS, resuspended in 400 μL PBS, and immediately analyzed on a NovoCyte 3000 flow cytometer (ACEA Biosciences, USA). DCF was excited by a 488 nm laser and detected at Em 530 ± 30 nm.

### 4.14. RT-qPCR Analysis

All primers were synthesized in the Laboratory of Biomedical Chemistry of ICBFM SB RAS (Novosibirsk, Russia) (Table S1, Supplementary Materials).

The cells were lysed with TRIzol Reagent, and total RNA was isolated following the manufacturer’s protocol. The RNA was quantified using a NanoDrop One^C^ Spectrophotometer (Thermo Fisher Scientific, USA). Subsequently, coding DNA (cDNA) was synthesized from the total RNA in a 40 μL reaction mixture containing 2 μg total RNA, 8 μL 5× RT buffer, 200 U M-MuLV-RH reverse transcriptase, and 1 μM random hexamer primers. Reverse transcription was performed at 42 °C for 60 min; termination of the reaction was at 70 °C for 10 min.

Quantitative PCR assay was performed using BioMaster SYBR Blue reagent kit (BioLabmix, Novosibirsk, Russia) according to the manufacturer’s recommendations. Primers were used at a final concentration of 400 nM, and the specificity of the amplification product was verified for each reaction by examination of the corresponding dissociation curve. The primer sequences are shown in Table S1 (Supplementary Materials). Amplification was performed with a CFX96™ Touch cycler as follows: 95 °C for 5 min, followed by 40 cycles of 95 °C for 10 s, 60 °C for 15 s, and 72 °C for 20 s. All reactions were carried out in triplicate. The expression of the studied genes was determined using the comparative cycle threshold method (2^−∆∆Ct^) and normalized to housekeeping genes *Hprt*. PCR data were analyzed using CFX Maestro Software Version 1.0 (Bio-Rad, Hercules, CA, USA).

### 4.15. Analysis of p65 Subunit Nuclear Translocation in Triterpenoid-Treated BM-MDSCs

Glass coverslips in 24-well plates were coated with poly-L-lysine solution for 20 min at room temperature. The solution was aspirated, and coverslips were washed twice with PBS. Triterpenoid-treated BM-MDSCs (4 × 10^5^ cells) were suspended in 500 µL complete RPMI-1640 medium supplemented with 100 ng/mL rmTNFα and seeded onto the coated coverslips for 4 h in 5% CO_2_ at 37 °C. Cells were fixed overnight in 4% paraformaldehyde in PBS at 4 °C, then permeabilized with 0.1% Triton X-100 and 0.5% BSA in PBS for 15 min at room temperature.

Following blocking with 0.05% Triton X-100 and 0.1% BSA in PBS for 30 min at room temperature, cells were incubated sequentially with primary anti-NF-kB p65/RelA (1:100 dilution, 30 min, room temperature), followed by Alexa Fluor 488-conjugated anti-IgG secondary antibody (1:500 dilution, 30 min, room temperature), and DAPI (1:1000 dilution, 5 min, room temperature). Slides were mounted and visualized using a confocal fluorescent microscopy LSM710 (Zeiss, Munich, Germany) using a plan-apochromat 63×/1.40 Oil DIC M27 objective. The obtained images were analyzed using ZEN software 2012 (Zeiss, Munich, Germany) and ImageJ software v. 1.53k (Wayne Rasband and contributors, NIH, USA). p65 nuclear translocation was assessed in MDSCs as the nuclear-to-cytoplasmic ratio of p65-related fluorescence intensity.

### 4.16. Molecular Docking

The blind molecular docking of triterpenoids with p65 was performed using AutoDock Vina v.1.2.5. The crystal structure of p65 in complex with DNA was obtained from the RCSB PDB database (PDB ID = 9BDW) and used as a docking template. DNA and water molecules were removed, after which polar hydrogen atoms and Gasteiger charges were added using AutoDock Tools 1.5.7. The three-dimensional structures of the triterpenoids were built in Marvin Sketch 5.12, and their geometry was optimized with Avogadro 1.2.0 using the MMFF94 force field. All rotatable bonds within the triterpenoids were allowed to be freely flexible. A grid box of 30 × 24 × 24 Å, centered at coordinates x, y, z = −37.588, 9.256, −27.31, respectively, was defined. The docking results were visualized using Discovery Studio Visualizer 17.2.0 (Dassault Systèmes, Vélizy-Villacoublay, France).

### 4.17. Statistical Analysis

The data were statistically analyzed using the two-tailed unpaired *t*-test, one-way ANOVA with post hoc Tukey’s test, and two-way ANOVA with Tukey’s multiple comparison tests. A *p*-value ≤ 0.05 was considered statistically significant. Statistical analysis was performed with GraphPad Prism v. 8.0.1 (GraphPad, San Diego, CA, USA).

## Data Availability

The original contributions presented in this study are included in the article/Supplementary Materials. Further inquiries can be directed to the corresponding author.

## Supplementary Materials

The following supporting information can be downloaded at https://www.mdpi.com/article/10.3390/ijms27020564/s1.

## Abbreviations

The following abbreviations are used in this manuscript:

MDSCs	Myeloid-derived suppressor cells
CTX	Cyclophosphamide
PMN-MDSCs	Polymorphonuclear myeloid-derived suppressor cells
GLZ	Glycyrrhizic acid
FM	Febroxolone methyl
M-MDSC	Monocytic myeloid-derived suppressor cells
Arg-1	Arginase-1
IDO	Indoleamine 2,3-dioxygenase
iNOS	Inducible nitric oxide synthase
IL-10	Interleukin-10
TGF-β	Transforming growth factor beta
ROS	Reactive oxygen species
NO	Nitric oxide
PD-L1	Programmed death-ligand 1
VEGF	Vascular endothelial growth factor
DOX	Doxorubicin
VCR	Vincristine
PNL	Prednisolone
5-FU	5-Fluorouracil
Treg	Regulatory T cells
COX-2	Cyclooxygenase-2
PGE2	Prostaglandin E2
CDOO-Me	Bardoxolone methyl
SM	Soloxolone methyl
BM	Bone marrow
