# Supplementary material for: Cyclophosphamide-Mediated Induction of Myeloid-Derived Suppressor Cells In Vivo: Kinetics of Accumulation, Immune Profile, and Immunomodulation by Oleanane-Type Triterpenoids
Source: Int J Mol Sci. 2026 Jan 6;27(2):564. doi: 10.3390/ijms27020564 (PMC12840754; doi:10.3390/ijms27020564)
Supplement: Supplementary file 1 [file ijms-27-00564-s001.zip › ijms-3975972-supplementary.pdf]

International Journal of *Molecular Sciences*

MDPI

Article

Cyclophosphamide-mediated induction of myeloid-derived suppressor cells *in vivo*: kinetics of accumulation, immune profile and immunomodulation by oleanane-type triterpenoids

Mona Awad [1,2], Aleksandra Sen'kova [1], Andrey Markov [1], Oksana Salomatina [3], Marina Zenkova [1] and Oleg Markov [1*]

1 Institute of Chemical Biology and Fundamental Medicine SB RAS, Academician Lavrentyev Ave. 8, 630090 Novosibirsk, Russia
2 Faculty of Natural Sciences, Novosibirsk State University, Pirogova str. 2, 630090 Novosibirsk, Russia
3 N.N. Vorozhtsov Novosibirsk Institute of Organic Chemistry SB RAS, Academician Lavrentyev Ave., 9, 630090 Novosibirsk, Russia
* Correspondence: markov_ov@1bio.ru (O. Markov); Tel.: +7(383)363-51-61

Supplementary materials

Table S1. List of primer sequences for RT-PCR.

Genes	Forward primer	Reverse primer
Hprt	5′-CCCCAAAATGGTTAAGGTTGC-3′	5′-AACAAAGTCTGGCCTGTATCC-3′
Arg1	5′-CCTTAGAGATTATCGGAGCG-3′	5′-CTCACGTCATACTCTGTTTC-3′
Nos1	5′- AAGGTCTACGTTCAGGACATC-3′	5′-AGAAATAGTCTTCCACCTGCT -3′
Pdl1	5′-CGGACTACAAGCGAATCAC-3′	5′-GTTTGTCCAGATTACCTCAGC-3′
Ido1	5′-CTTGTGGCTAGAAATCTGC-3′	5′-GCTGTAACCTGTGTCCTC-3′
Tnfa	5′-CCCTCCAGAAAAGACACCATG -3′	5′-GCCACAAGCAGGAATGAAG -3′
Il-10	5′-GTCATTTTCTGCCTCATCCT -3′	5′-GAGCCCTTTTAGACCTTTT -3′
Mmp9	5′- ACCTGAAAACCTCCAACCTC-3′	5′- TCGAATGGCCTTTAGTCTG-3′
Tgfb1	5′-CCTGAGTGGCTGTCTTTTG-3′	5′-CGTGGAGTTTGTTATCTTTGC-3′

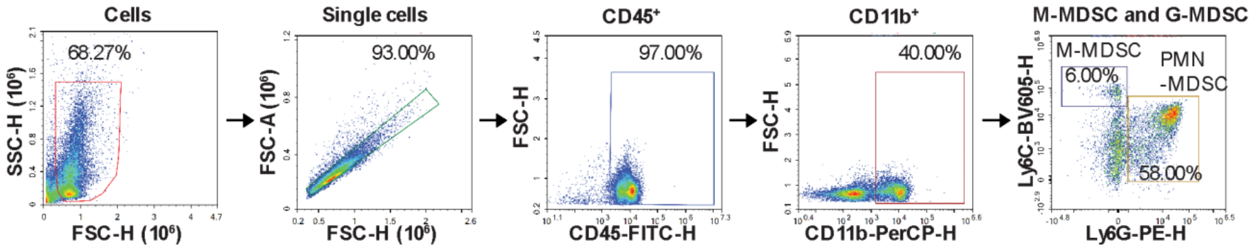

Figure S1. Gating strategy for the identification of mouse MDSC subsets in the peripheral blood and spleen.

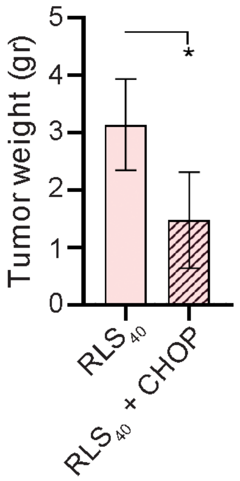

Figure S2. RLS_{40} tumor regression in RLS_{40}-bearing CBA mice after CHOP chemotherapy. Analysis was performed on day 23 after tumor transplantation (day 10 after the third round of CHOP). Tumor weight was calculated by subtracting the weight of the intact contralateral leg from the weight of tumor-bearing leg. Data represent two independent biological experiments (n = 4) and are presented as mean ± SD. Statistical analysis was performed by two-tailed unpaired t-test. *$p < 0.05$.

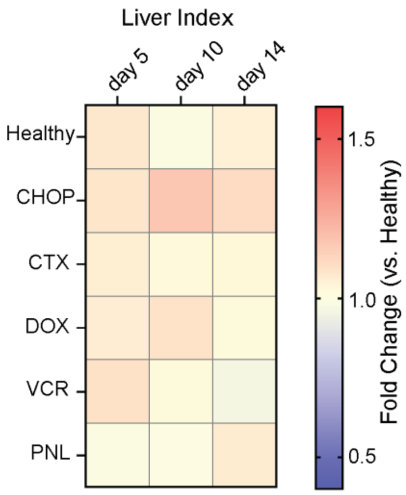

Figure S3. Dynamic changes of liver indices induced by either CHOP regimen or individual CHOP components in healthy CBA mice. Heat maps depicting liver indices of experimental mice, showing organ-to-body weight ratios normalized to healthy control. Data represent two independent biological experiments (n = 5).

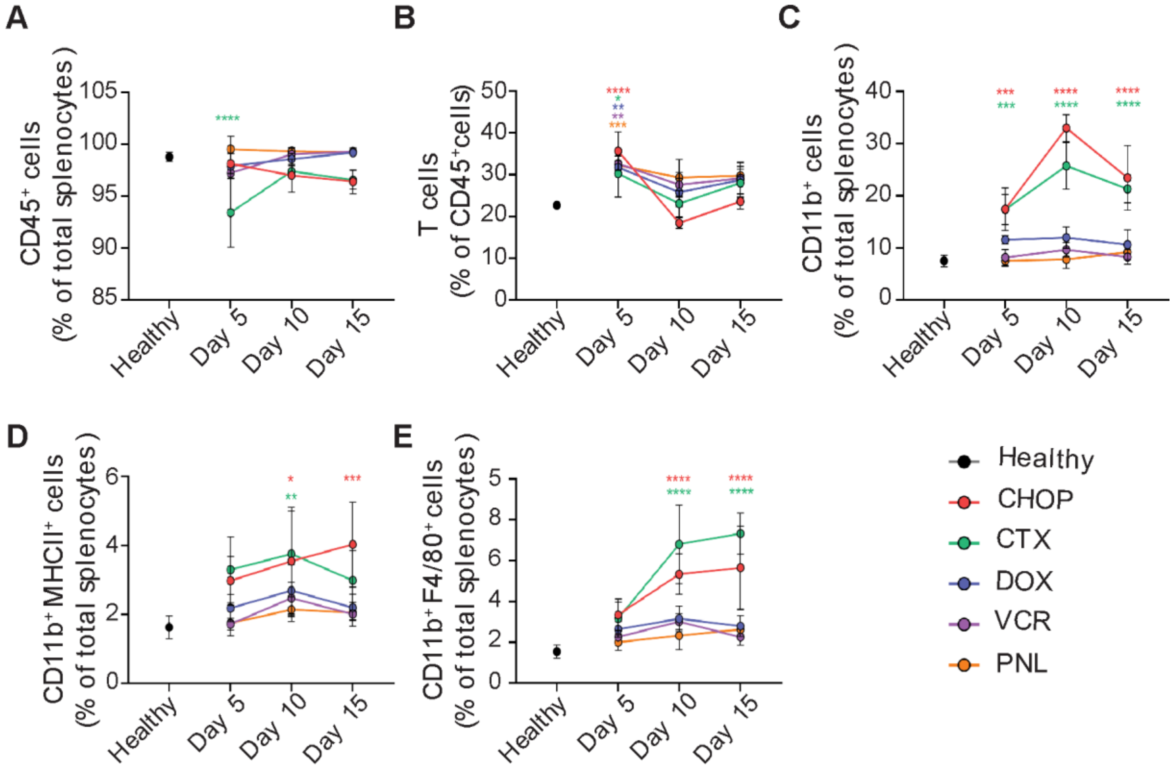

Figure S4. The contribution of individual CHOP components to immune cell alterations in spleens of healthy mice. Male CBA mice received i/p CHOP polychemotherapy or its individual components CTX, DOX, VCR, or PNL three times with four-day intervals. Splenocytes were analyzed by flow cytometry at days 5, 10, and 15 post-treatment to determine the percentages of (A) CD45⁺ cells, (B) CD3⁺ T cells, (C) CD11b⁺ cells, (D) CD11b⁺MHCII⁺ cells, and (E) CD11b⁺F4/80⁺ cells. Data are presented as mean ± SD (n =5). Statistical analysis by one-way ANOVA with Tukey's post hoc test; *p < 0.05, **p < 0.01, ***p < 0.001, ****p < 0.0001.

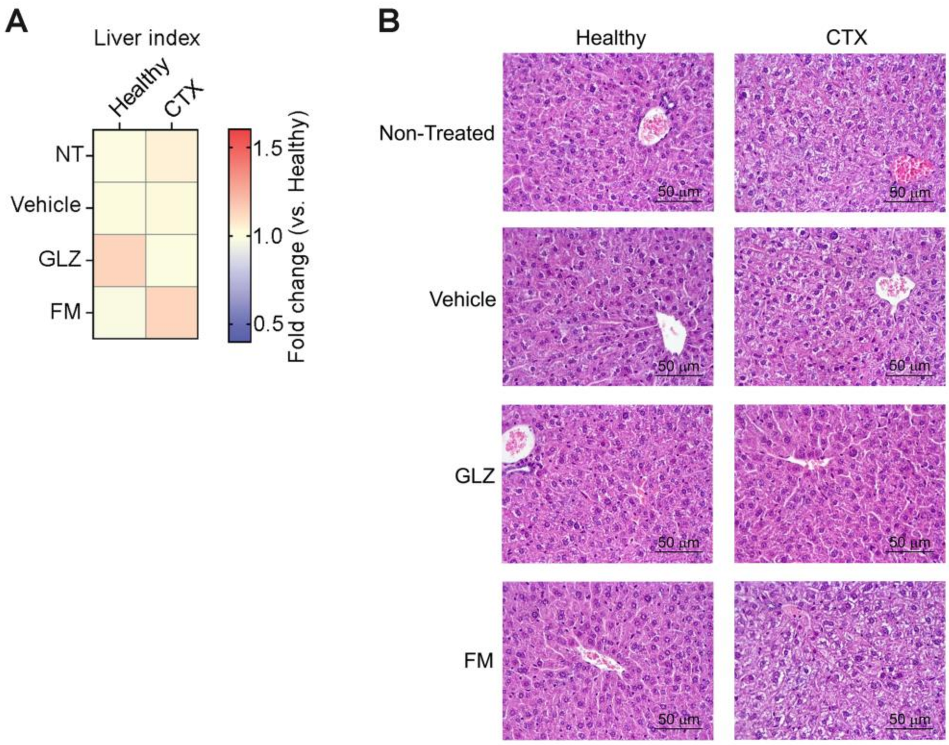

Figure S5. Effects of GLZ and FM triterpenoids on liver of CTX-treated healthy mice. (A) Heat maps depicting liver indices of experimental mice, showing organ-to-body weight ratios normalized to healthy control. Data represent two independent biological experiments (n = 5). (B) Structural changes in the liver tissue of healthy and CTX-treated mice after GLZ and FM administration. Hematoxylin and eosin staining. Original magnification ×400.

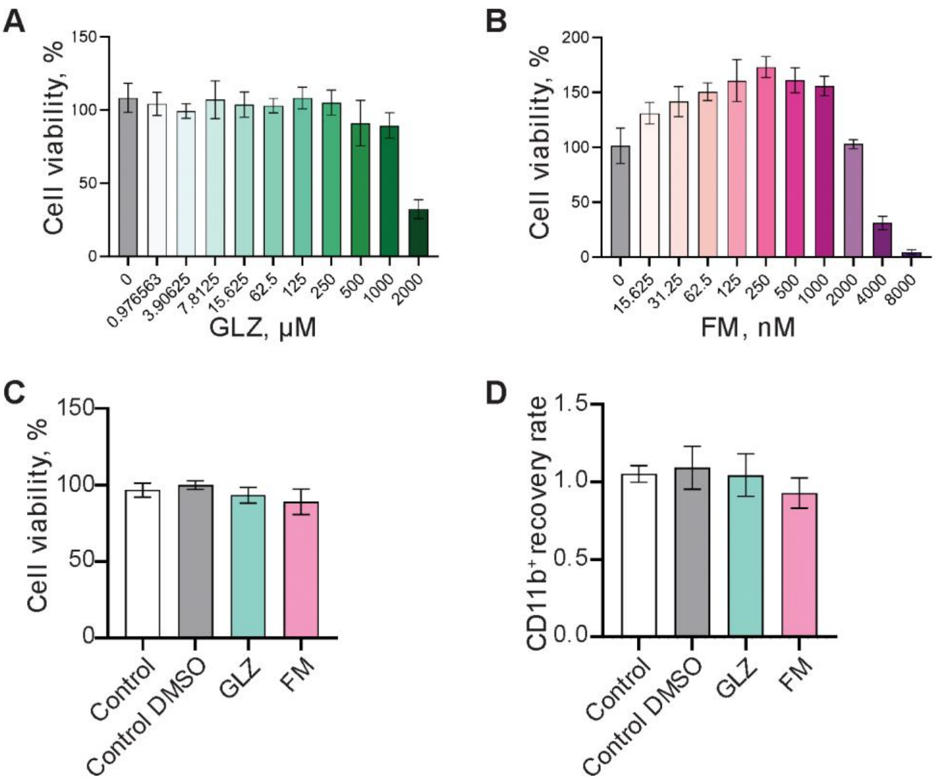

Figure S6. Effects of GLZ and FM on the viability of *in vitro* generated BM-derived MDSCs. (A, B) WST-1 assay. Bone marrow cells were differentiated into MDSCs in the presence of various concentrations of (A) GLZ or (B) FM for 4 days. Cell viability expressed as the percentage of viable cells relative to DMSO-treated control cells, untreated control was set as 100%. (C) Trypan blue exclusion assay. Viability of BM-derived MDSCs generated in the presence of DMSO (0.5 μL/mL), 250 μM of GLZ, or 250 nM of FM. (D) The recovery rate of BM-derived MDSCs, representing the ratio of the total yield of BM-derived $CD45^+CD11b^+$ cells to the initial number of seeded BM cells (combined trypan blue and flow cytometry data). Data are presented as mean ± SD from three independent experiments.
